# Changes in Protonation Sites of 3-Styryl Derivatives of 7-(dialkylamino)-aza-coumarin Dyes Induced by Cucurbit[7]uril

**DOI:** 10.3389/fchem.2022.870137

**Published:** 2022-04-14

**Authors:** Jackson J. Alcázar, Edgar Márquez, Luis García-Río, Agustín Robles-Muñoz, Angélica Fierro, José G. Santos, Margarita E. Aliaga

**Affiliations:** ^1^ Facultad de Química y de Farmacia, Pontificia Universidad Católica de Chile, Santiago, Chile; ^2^ Departamento de Química y Biología, Facultad de Ciencias Exactas, Grupo de Investigaciones en Química y Biología, Universidad Del Norte, Barranquilla, Colombia; ^3^ Departamento de Química Física, Centro de Investigación en Química Biológica y Materiales Moleculares (CIQUS), Universidad de Santiago, Santiago, Spain

**Keywords:** heterocyclic nitrogen protonation, 7-dialkylamino-aza-coumarin dyes, protonation induced by cucurbit[7]uril, molecular recognition by cucurbit[7]uril, spectral behaviour of protomers

## Abstract

The incorporation of a guest, with different basic sites, into an organized system (host), such as macrocycles, could stabilize, detect, or promote the formation of a certain protomer. In this context, this work aimed to study the influence of cucurbit[7]uril (CB7) on dyes such as 7-(dimethylamino)-aza-coumarins, which have more than one basic site along their molecular structure. For this, three 3-styryl derivatives of 7-(dialkylamino)-aza-coumarin dyes (**SAC1-3**) were synthesized and characterized by NMR, ESI-HRMS and IR. The spectral behaviour of the SACs in the absence and presence of CB7 was studied. The results showed large shifts in the UV-vis spectrum in acid medium: a hypsochromic shift of ≈5400 cm^−1^ (**SAC1-2**) and ≈3500 cm^−1^ (**SAC3**) in the absence of CB7 and a bathochromic shift of ≈4500 cm^−1^ (**SAC1-3**) in the presence of CB7. The new absorptions at long and short wavelengths were assigned to the corresponding protomers by computational calculations at the density functional theory (DFT) level. Additionally, the binding mode was corroborated by molecular dynamics simulations. Findings revealed that in the presence of CB7 the heterocyclic nitrogen was preferably protonated instead of the dialkylamino group. Namely, CB7 induces a change in the protonation preference at the basic sites of the SACs, as consequence of the molecular recognition by the macrocycle.

## Introduction

The structure of a molecule in solution depends on the intrinsic properties of the molecule itself as well as its interactions with the surrounding solvent. The properties of a charged molecule, including its structure and reactivity, are influenced by the location of the charge, such as the protonation site(s) (Cox et al., 1996; Graton et al., 2002; Tu, 2006; Wu and McMahon, 2007; Bull et al., 2017). The existence of so-called protomers (Demireva and Armentrout, 2021), molecular isomers that only differ in the site of protonation, is therefore of great importance for both fundamental research and applications.

The site of protonation in solution of a compound depends not only on their relative intrinsic strengths but also on the different stabilizations by the solvent of the different protonated forms (Demireva and Armentrout, 2021). As an example, protonation of *p*-aminobenzoic acid derivatives (Bagno and Scorrano, 2000; Demireva and Armentrout, 2021) which has been widely studied in solution and gas-phase. Although the amine nitrogen is the most basic site in aqueous environments, the carbonyl oxygen becomes energetically favorable for protonation when the relative permittivity decreases.

The solvation shell of a solute dissolved in a solvent mixture is generally selectively enriched in one cosolvent. When this happens, the solute is said to be preferentially solvated, and this has an obvious bearing on all its solvation-related properties, since its immediate surroundings may be substantially different from the bulk solution. Preferential solvation may be achieved by formation of host:guest complexes. Guest incorporation into an organized system (supramolecular receptor) originates a nonhomogeneous solvation shell in such a way that different parts of the substrate are sensing different microenvironments (Pereira-Vilar et al., 2016).

In particular, synthetic organic dyes are widely used as pigments in many commercial products (Ziarani et al., 2018). These dyes have been widely studied in supramolecular chemistry, either by analyzing fundamental chemical interactions, tautomeric equilibrium related to guest solvation or as components of assemblies in different applications (Dsouza et al., 2011; Ziarani et al., 2018).

Among organic dyes, those containing 7-dialkylamino-2*H*-chromen-2-one scaffolds with 3-substituted electron acceptor moieties have had numerous applications, due to their optical and fluorescent properties: large Stokes shifts, emissions in the visible light range, and high quantum yields (Cao et al., 2019). However, they usually have short wavelength (UV) excitation, making them less than ideal for cell assays or imaging (Fu and Finney, 2018). This problem seems to be solved by replacing the CH group in position 4 of the scaffold with a nitrogen atom, allowing it to absorb longer wavelengths (Trebaul et al., 1987). This new family of structures is known as 7-(dialkylamino)-4-azacoumarin, also called aminobenzoxazinone.

The styryl derivatives of 7-(dimethylamino)-aza-coumarin in different solvents, were photophysically studied by Fery-Forgue (Fery-Forgues et al., 1992), demonstrating the importance of the main chromophore, 7-(dialkylamino)-aza-coumarin moiety, and the *p*-substituted styryl moiety. The authors reported that some of these derivatives could potentially be used to probe hydrophobic regions in biological media.

Accordingly, the 7-(dialkylamino)-aza-coumarins, in general, have been shown to possess similar, or more suitable photophysical properties than their analogue coumarin (Le Bris, 1985; Trebaul et al., 1987; Fery-Forgues et al., 1992). These derivatives have been used in the detection of analytes in biological systems such as bisulfite/sulfite (Agarwalla et al., 2016; Li et al., 2017; Chen et al., 2020; Lu et al., 2020), biothiols (Liu et al., 2013; Agarwalla et al., 2018), arginine (Yu et al., 2017), cysteine (Hu et al., 2011), cyanide and hypochlorite ion (Agarwalla et al., 2015; Fan et al., 2015), among others (Depierreux et al., 1990; Monsigny et al., 1990; Fan et al., 2012), and some have been reported with excellent cell imaging. For this purpose, 7-(dialkylamino)-aza-coumarins are designed with receptors at carbon 3, inspired by their coumarin analogues (Kwon et al., 2011; Anila et al., 2015; Han et al., 2020). Recently, the importance of designing new molecular systems that facilitate the participation of heterocyclic nitrogen as ionophores for heavy metal ion recognition has been discussed (Nagarajan et al., 2021). These designs can be associated with changes in the scaffold of the *N*-heterocyclic molecule which, using supramolecular host-guest systems, could act as a differential metal ion sensing (Xu et al., 2010; Selvan et al., 2018), or in a synergetic binding mode (Xu et al., 2010).

Among the hosts capable of harboring molecules of an aromatic nature, cucurbit[7]uril (CB7) stands out (Barrow et al., 2015). This macrocycle has been used in different studies due to its structural dimensions and its high affinity for electron-poor sites (Assaf and Nau, 2015; Macartney, 2018). In this context, dialkylamino substituted coumarins or other dialkylamino substituted chromophores have played a leading role in several studies in the presence of CB7, forming stable inclusion complexes on the side of the dialkylamino group in its protonated or deprotonated form (Barooah et al., 2012, 2014; Manna and Chakravorti, 2015; Aliaga et al., 2017; Basílio et al., 2017; Jana et al., 2019; Paudics et al., 2020; Alcázar et al., 2021). On the other hand, the pyridinium group has also shown a remarkable affinity for CB7 (Manna and Chakravorti, 2015; Jana et al., 2019; Paudics et al., 2020). The interaction of CB7 with these two groups can substantially increase their p*K*a values by stabilizing the conjugated acid (Basarić et al., 2015; Basílio et al., 2017; Behera and Krishnamoorthy, 2017).

**FIGURE 1:**
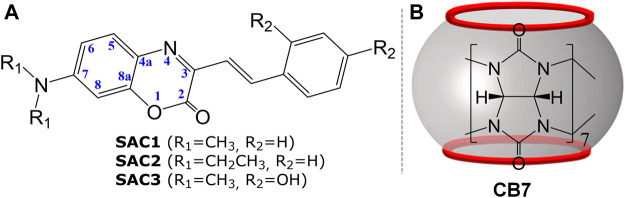
Representation of: **(A)** Series of 3-styryl derivatives of 7-(dialkylamino)-aza-coumarins (**SAC1-3**) and **(B)** cucurbit[7]uril (CB7).

Therefore, of special interest is the influence of CB7 on organic synthetic dyes with more than one basic site, as occurs in the derivatives of 7-(dialkylamino)-aza-coumarins where the 7-dialkylamino group and the heterocyclic nitrogen could be competing for the acceptance of a proton in an acid medium. In this context, this work combined experimental and theoretical study of the spectrophotometric and fluorometric characterization of the protomeric forms of the 3-styryl derivatives of 7-(dialkylamino)-aza-coumarins (**SAC1-3**) in the absence and presence of CB7 (Figure 1).

## Results and Discussion

### Synthesis of 3-Styryl Derivatives of 7-(dialkylamino)aza-coumarins (SAC)

**SCHEME 1:**
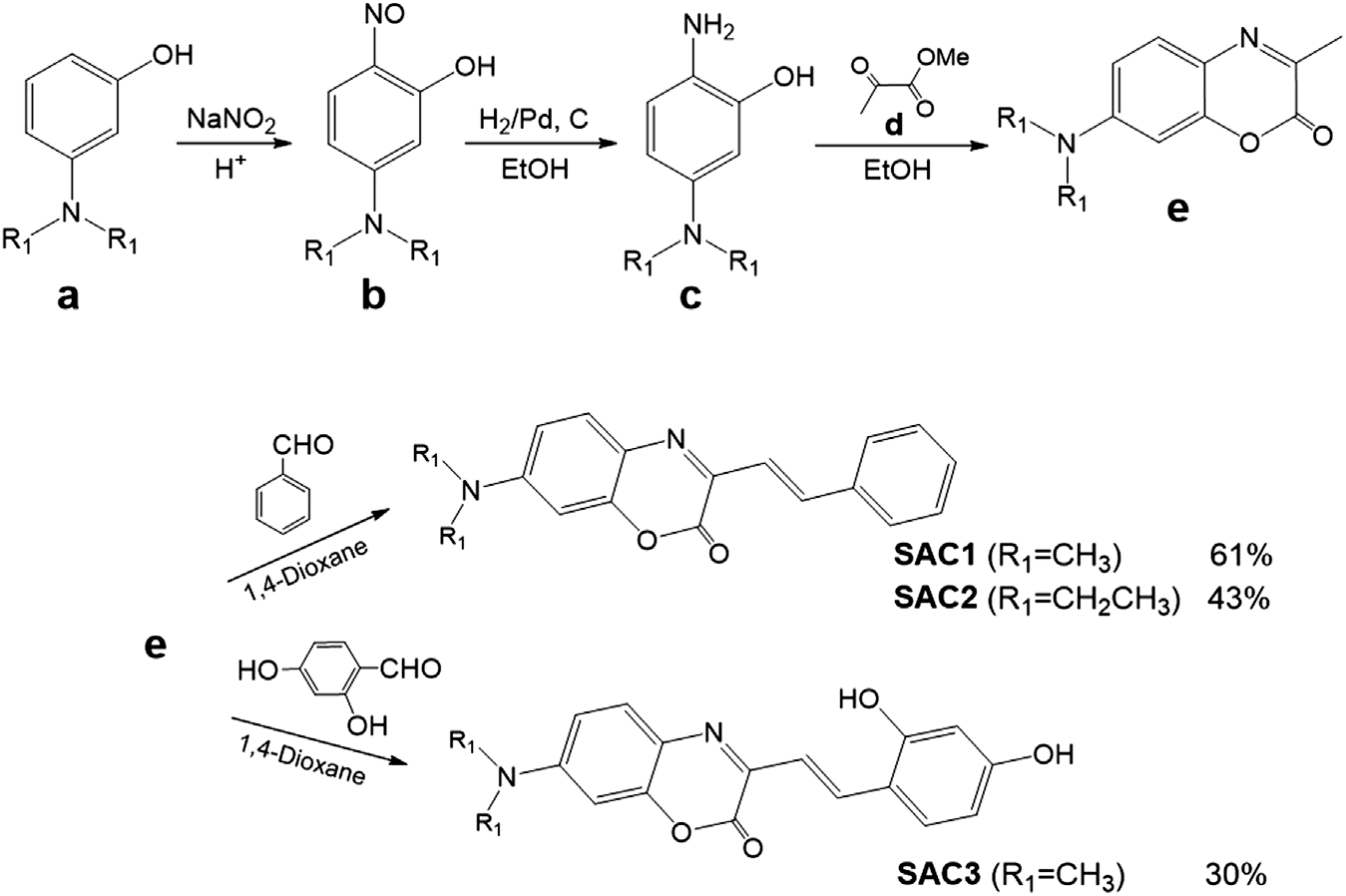
Synthetic routes to obtain the SAC series (**SAC1-3**).

In order to carry out the synthesis of SACs, the original methodology described by Bris *et al.* was modified (as shown in Scheme 1). Among the most relevant modifications was the substitution of acetic anhydride as solvent by 1,4-dioxane, to obtain the hydroxylated SAC (**SAC3**) from 7-(dimethylamino)-3-methyl-aza-coumarin and 2,4-dihydroxybenzaldehyde, thus avoiding the acetylation reaction. Since the reaction occurs at temperatures above the boiling point of dioxane, a reaction tube was used. This new methodology was also tested for the synthesis of the substrates **SAC1** and **SAC2** (not hydroxylated), but the yields were lower than those reported by Bris *et al.*, (Le Bris, 1985).

The SAC homologous series were synthesized according to Scheme 1, see synthesis details and characterization by NMR (Supplementary Figures S1–S6), IR and ESI-HRMS in experimental section.

### pKa Values for SAC1-3 From Titration Data: An Experimental and Density Functional Theory Study

pKa values for **SAC1-3** were determined by UV-vis spectroscopy using the acid-base titration method in a methanolic solution (3:7 v/v) followed by a fit of the titration data to equation Eq. 1 (see experimental section).

**FIGURE 2:**
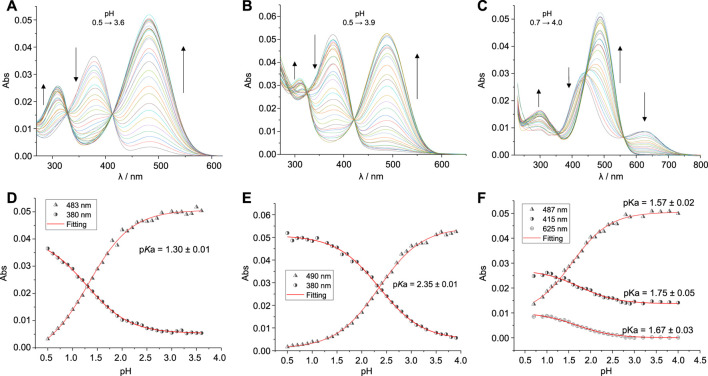
Top*:* Modifications of the absorption spectra by acid-base titrations at different pH values (0.5–3.9) for **SAC1-3** (1.5 μM; T = 25.0°C) in methanol/water, 3/7 (v/v): **(A) SAC1**, **(B) SAC2** and **(C) SAC3**. Bottom: Acid base titration curves at different absorption wavelengths (represented by different symbols) and the fitting of Eq. 1 to experimental data (represented by red continuous lines): **(D) SAC1**, **(E) SAC2** and **(F) SAC3**.

As shown in Figure 2, the **SAC1-3** compounds exhibited a similar *λ*_max_ (around 488 nm) at pH close to 4, while at pH close to 0.5 *λ*_max_ appeared at 380 nm for the compounds **SAC1** and **SAC2**. Interestingly, **SAC3** presented two *λ*_max_ (431 and 625 nm) at pH close to 0.7, suggesting two possible protonation sites.

The p*K*a determined for the protonated form of **SAC2** is 2.35 (Table 1). This p*K*a value is similar to that reported for the diethylaminio group for series of 7-diethylamino-substituted coumarins (Kirpichënok et al., 1991; Patalakha et al., 1991). This suggests the p*K*a obtained for **SAC2** is associated with the diethylamino group. Consequently, it can be deduced that the p*K*a found for **SAC1** (p*K*a = 1.30) is linked to the dimethylamino group. This difference in p*K*a between both groups (dimethyl- and diethylamino) was also observed in derivatives of dialkylamino substituted chalcones (Basílio et al., 2017).

**TABLE 1:** Experimental and theoretical p*K*a values and maximum absorption lengths of the neutral (*λ*_abs_) and protonated (λ_abs_^H^) SACs.^a^

Substrates	Experimental			Theoretical	
	p*K*a	λ_abs_^H^ (nm)	λ_abs_ (nm)	λ_abs_^H^ (nm)	λ_abs_ (nm)
SAC1	1.30 ± 0.01	380	483	383^b^	469
SAC2	2.35 ± 0.01	380	493	384^b^	473
SAC3	1.75 ± 0.05	431	487	424^b^	480
	1.67 ± 0.03	625	−	634^c^	−

a In methanolic solution (3:7 v/v) at 25.0°C.

b Mono-protonated substrate on the dialkylamino nitrogen.

c Mono-protonated substrate on the heterocyclic nitrogen.

In this context, DFT calculations confirmed that this protonation equilibrium corresponds to the dialkylamino group. In fact, a *λ*_max_ just above 380 nm corresponds to **SAC1** and **SAC2** when protonated on the dialkylamino nitrogen (Table 1). Hence, it can be reasonably deduced that the obtained p*K*a for **SAC1** and **SAC2** correspond to the respective dialkylammonium group, where the highest value of p*K*a for **SAC2** is a consequence of greater stabilization by hyperconjugation of the substituent *N,N*-diethyl compared to the substituent *N,N*-dimethyl of **SAC1**.

On the other hand, the band corresponding to the protonation of the dimethylamino group for the **SAC3** compound is centered at 431 nm (not at 380 nm, as in **SAC1-2**). This would be due to the presence of the hydroxyl groups in the phenyl group. Furthermore, the same bathochromic shift was obtained through DFT calculations, as shown in Table 1. Moreover, the band centered at 625 nm for the **SAC3** compound was reproduced by DFT calculations (with a difference of 9 nm) when **SAC3** was protonated only in the heterocyclic nitrogen. Lastly, UV-vis theoretical calculations for di-protonated **SAC3** did not produce a *λ*_max_ beyond 467 nm, ruling out the di-protonated form (Supplementary Table S1). Therefore, it can be deduced that, with a pH close to 0.7, **SAC3** presents two non-consecutive protonations: one on the nitrogen of the dimethylamino group (p*K*a = 1.75) and the other on the heterocyclic nitrogen (p*K*a = 1.67).

### Feasibility for the Formation of Complexes Containing SAC Dyes and the cucurbit[7]uril (CB7): A Molecular Dynamic Study

Based on the equilibrium between the protonated form and non-protonated form of SAC dyes, we studied the feasibility of including this organic dyes into the hydrophobic macrocycle of CB7, by use of molecular dynamic simulations. Due to complexation-induced p*K*a shifts, it is reasonable to consider the protonation of *N*,*N*-dialkylamino moiety of the dyes inside of CB7. However, illustrative data of molecular dynamic simulations for non-protonated form of SAC within CB7, were also carried out.

**FIGURE 3:**
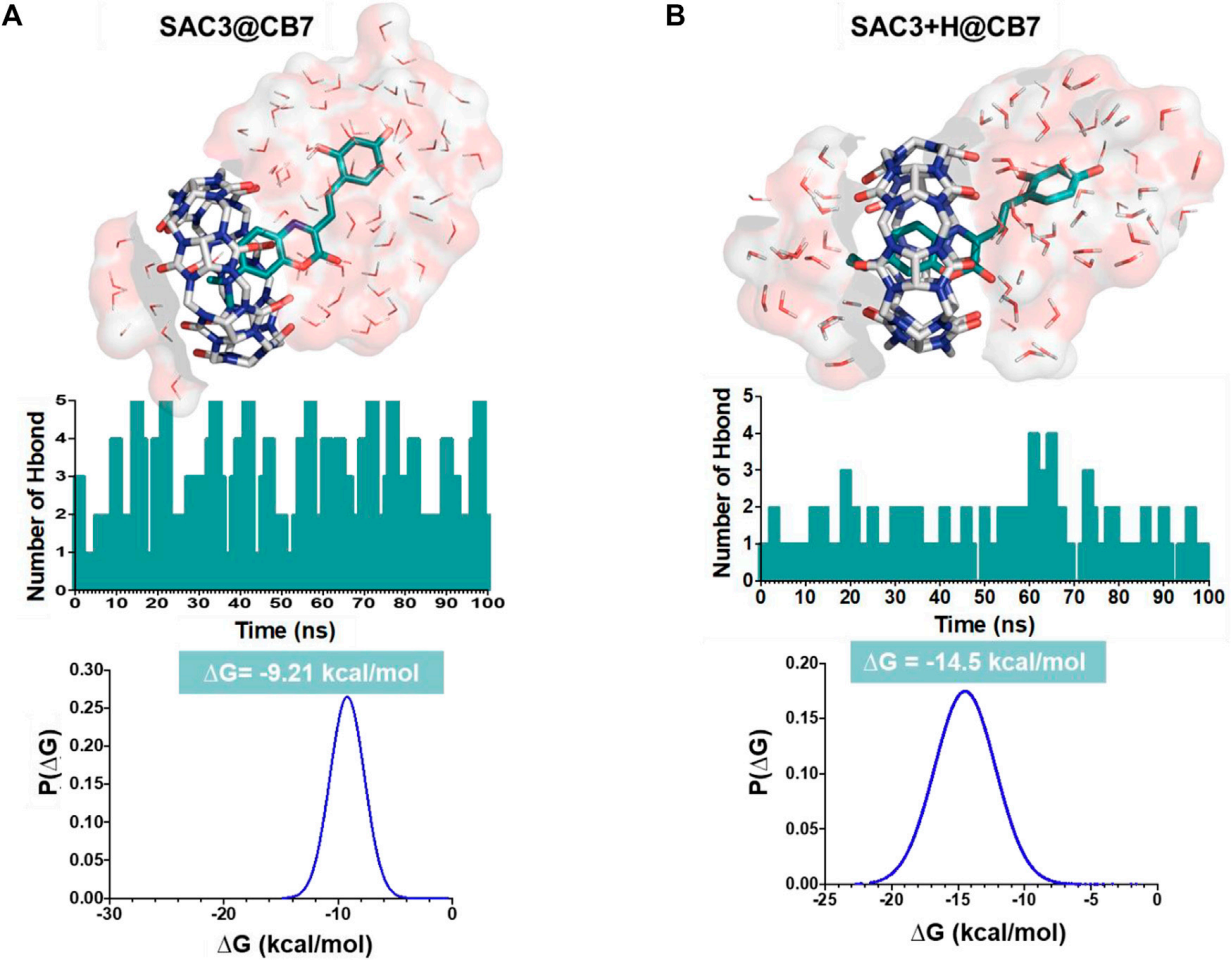
Final conformations from molecular dynamic simulations of the CB7 containing complexes with the non-protonated form of **SAC3 (A)** and the protonated form (on the dialkylamino nitrogen) of **SAC3 (B)**. In the middle panel, histograms of the number of hydrogen bonds during the simulation are displayed. On the bottom panel, a free energy distribution from MM/PBSA calculations is shown.

Figure 3 shows the results obtained from molecular dynamic simulations for the plausible conformations of representative complexes between CB7 and **SAC3** (non-protonated and protonated and forms). The non-protonated form of **SAC3** in CB7 (Figure 3A) shows stabilization by a hydrogen bond network involving a permanent exposure of the aza-coumarin moiety at the solvent during the molecular dynamic simulation. On the other hand, the protonated form of **SAC3** (Figure 3B) localizes the aza-coumarin fragment immersed into the non-polar segment of the macrocycle while the alkylammonium segment, which during the simulation remains close to the CB7 portal, is stabilized by water molecules. In addition, a Coulombic interaction generated by the protonated nitrogen in *N,N*-diethyl ammonium group and the negative electronic density from the carbonyl groups from the macrocycle during 100 ns (Supplementary Figure S7) was observed. Thus, our results show that, whilst SAC3@CB7 is stabilized by a hydrogen bond network, the charge on the ammonium group in SAC3+H@CB7 contributes significantly to the final binding free energy (Figure 3, bottom).

Interestingly, all results obtained provide evidence of the formation and stabilization of these inclusion complexes *via* a favorable free energy distribution (−14.5 kcal mol^−1^ and −9.2 kcalmol^−1^ for protonated and non-protonated forms, respectively).

### Inclusion Effects of SAC1-3 dyes Into CB7 Host

**FIGURE 4:**
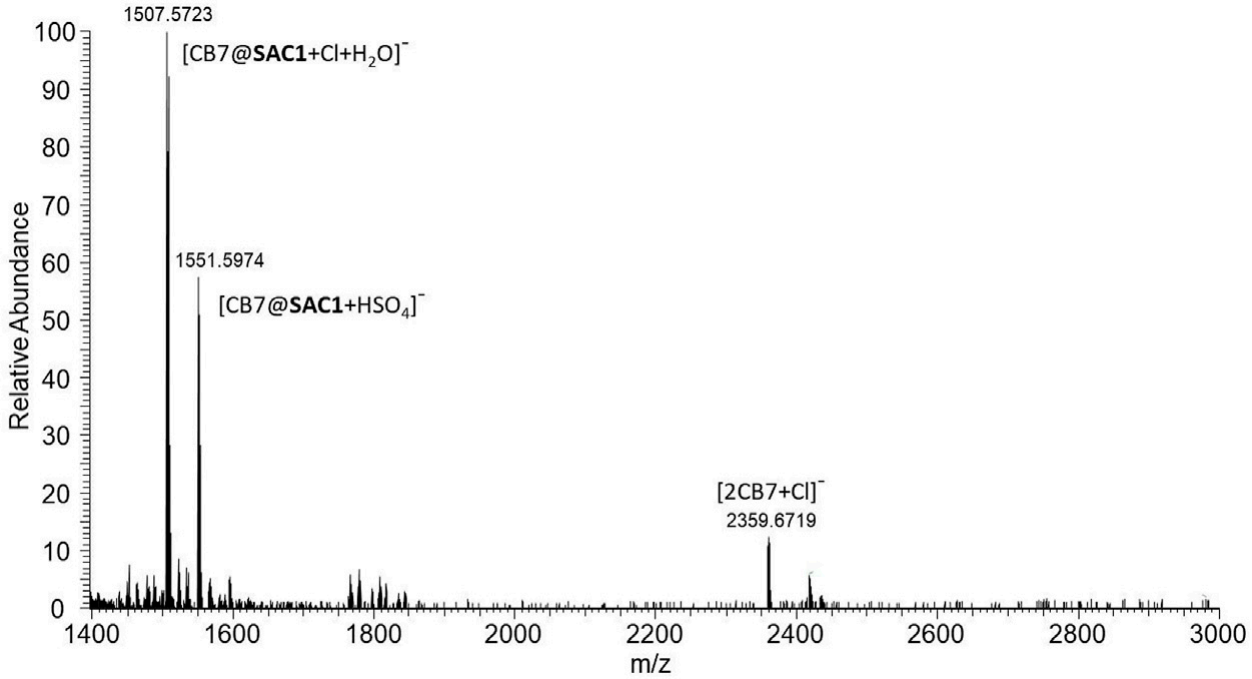
ESI-HRMS (Ionization voltage: 3.5 kV and negative polarity) for 1.5 µM of **SAC1** in the presence of 1mM of CB7, dissolved in a methanolic solution at 30% (v/v) and pH 2.5. Scan parameters: Resolution: 140000, AGC target: 1 × 10^6^, Max. inject time: 200. HESI source: Sheath gas flow: 25, Aux gas flow rate: 3, Sweep gas flow rate: 0, Capillary temp.: 250°C, S-lens RF level: 100, Heater temp: 100°C.

To study the effect on the physicochemical properties of the inclusion of **SAC1-3** in CB7, combined experimental (UV-vis spectroscopy and HRMS) and theoretical studies (DFT) were carried out. As shown in Supplementary Figure S8, the **SAC1** dye presented a new band at longer wavelengths (*λ*_max_ = 611 nm) in presence of CB7, indicating the formation of an inclusion complex. In fact, the mass spectrum suggests the formation of 1:1 stoichiometry complexes (Figure 4 and Supplementary Figure S9).

**FIGURE 5:**
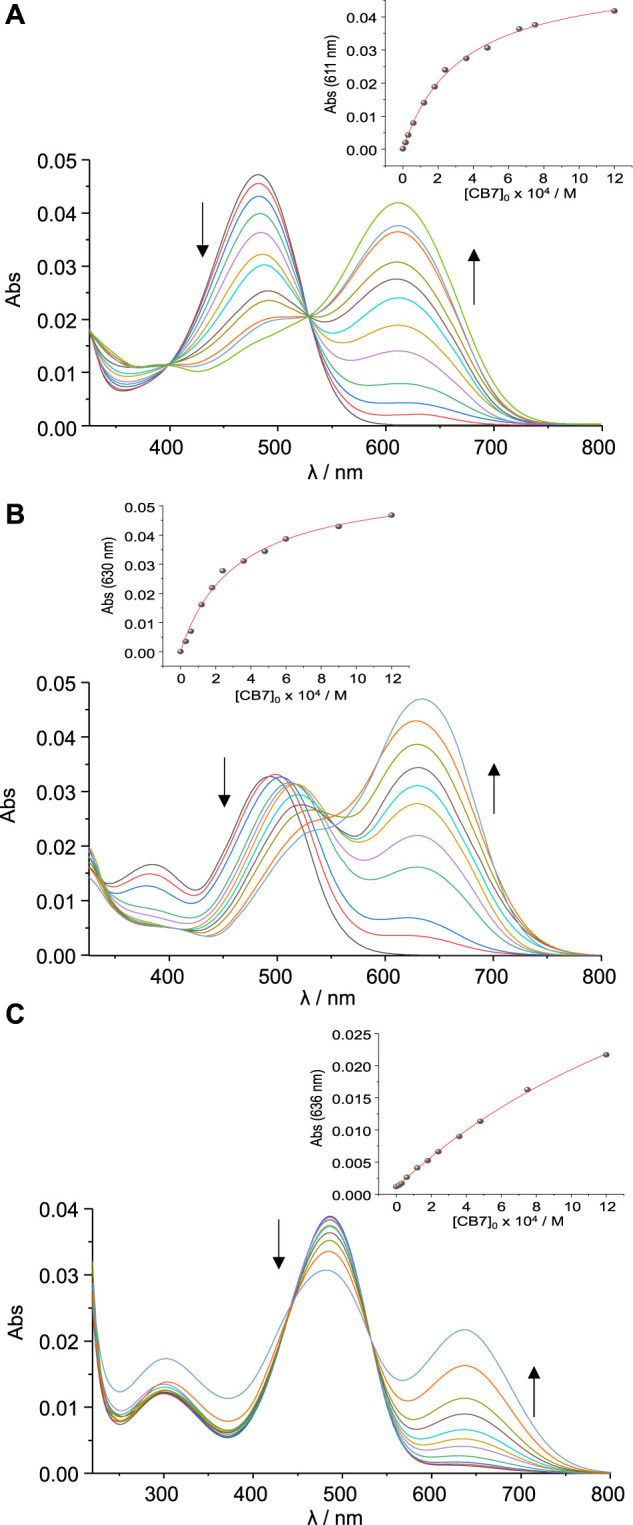
Modification of the UV-vis spectra by titrations with CB7 for the SAC compounds (1.5 μM at pH 2.5 in 30% methanol and T = 25.0°C): **(A) SAC1**, **(B) SAC2** and **(C) SAC3**.

As shown in Supplementary Figure S8B, the intensity of the band at 611 nm increases as the pH decreases. From this relation it is inferred that the formed complex would be favored by the protonation of the substrate. Figure 5 depicts the titration curves with CB7 for **SAC1-3**, showing similar spectroscopic behavior for all compounds at wavelengths close to 620 nm and confirming the formation of complexes with the macrocycle.

The band at 611 nm (Figure 5A) could be associated with the protonation, induced by CB7, of the heterocyclic nitrogen of **SAC1**, from the neutral species. In this way, the shift would be 4447 cm^−1^ (483 nm → 611 nm), in line with the literature for proton transfer reactions (Mohanty et al., 2006; Wu and Isaacs, 2009; Barooah et al., 2012, 2014) or iminium ion formation reactions (Zhang et al., 2020). For the free compound **SAC3**, the protonation established for the heterocyclic nitrogen was previously discussed and assigned to the band at 625 nm (Figure 2C), which is a *λ*_max_ close to that observed for the **SAC1** in the presence of CB7 (Figure 5A).

Binding constants were estimated by non-linear regression approach from absorption titrations fitting the experimental data to a simple 1:1 stoichiometry model (see experimental section). The results indicate that substrates **SAC1** and **SAC2** present similar binding constants (≈3000 M^−1^; Supplementary Table S2). The **SAC2** compound exhibited an additional gradual shift, in relation to UV-vis spectral of **SAC1,** from the band located at 493 nm towards ≈540 nm.

The mass spectrum for **SAC2** in the presence of an excess of CB7 suggests only the formation of a 1:1 stoichiometry complex (Supplementary Figure S9), so it is unlikely that such shift is associated with a second 2:1 stoichiometry complex. Unfortunately, due to the high insolubility of the substrates (**SAC1-3**) in the experimental conditions (30% MeOH, T = 25.0°C), the analysis by NMR of the structure of the formed inclusion complexes could not be carried out.

**SCHEME 2:**
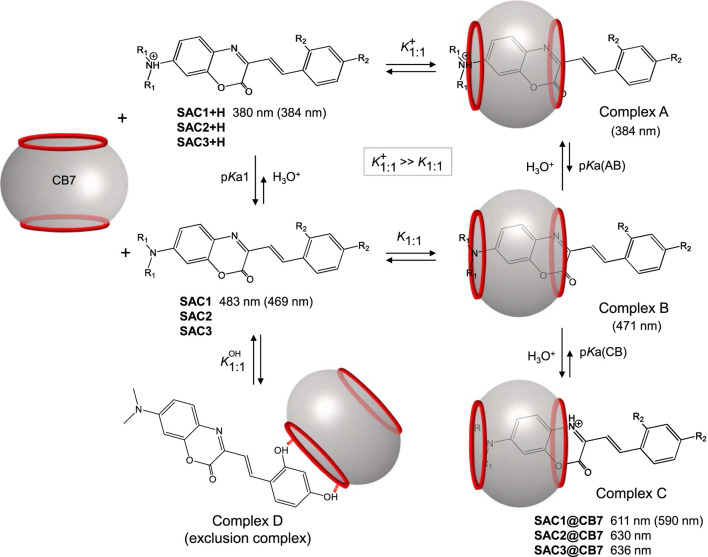
Representation of the proposed mechanism for the formation of the inclusion complex between CB7 and **SAC1-3** at pH 2.5. The values in nm are the maximum absorption wavelengths. The values in parentheses are the theoretical wavelengths calculated by DFT theory for **SAC1** and its host-guest complexes.

It is important to mention that the fitting of binding constants was satisfactorily obtained from 1:1 stoichiometry model. In the presence of CB7, **SAC3** presented a constant (412 M^−1^) which is 7.2 times lower than the estimated for **SAC1** and **SAC2**. Considering all above mentioned the binding mechanism illustrated in Scheme 2 is proposed.

In the proposed mechanism (Scheme 2), complexes A and C would be favored over complex B, due to ion-dipole interactions and the formation of hydrogen bonds, which allow protonated species to form complexes ≈1000 times more stable than the complexes formed by neutral substrates (Moghaddam et al., 2011). Complex C would be formed due to the protonation of heterocyclic nitrogen from complex B and would be responsible for the absorption band observed in the region from 610 to 640 nm. This protonation would be unfavored at pH 5.3 and 10, as shown in Supplementary Figure S8B. In addition, complexes A and B would maintain the same optical properties of the free substrate. The DFT calculations (theoretical values in parentheses) support this deduction, reproducing the *λ*_max_ observed experimentally (see Scheme 2).

On the other hand, the gradual shift from 493 nm towards ≈540 nm (Figure 5B), would be related to the neutral complex formed (complex B), since at lower pH values (specifically at pH 0.5) such shift was not observed (Supplementary Figure S10). In fact, this shift corresponds to 1902 cm^−1^, similar to that observed in non-protonated diethylamino substituted chalcones (2235 cm^−1^) (Basílio et al., 2017).

The explanation as to why this shift is only observed with the **SAC2** substrate and not with the **SAC1** substrate (Figure 5) could lie in a difference of p*K*a values for the equilibrium between complex A and B (p*K*a(AB), Scheme 2). For **SAC1**, this equilibrium should be more displaced towards complex A compared to when the guest is **SAC2**. In this sense, the concentration of complex B (when the guest is **SAC1**) should not be sufficient to produce a UV-vis signal like that observed in the case of **SAC2** (Figure 5B). This is supported by that reported by Basílio *et al.,* where the observed p*K*a for the complex formed between chalcones and CB7 is much higher when the substituent present in the substrate is dimethylamino (p*K*a = 6.22) in comparison with the substituent diethylamino (p*K*a = 4.71) (Basílio et al., 2017).

Lastly, the lower *K*_1:1_ binding constant value observed for the **SAC3** substrate compared to that observed for **SAC1** and **SAC2** (in the presence of CB7), may be due to the formation of an additional complex (complex D) as a result of an exclusion process between **SAC3** and CB7 (on the phenyl group side). Unfortunately, fitting the data obtained to the mechanism proposed in Scheme 2 is not possible without knowing at least all the p*K*as involved. Therefore, the estimated binding constant *K*_1:1_, in the presence of CB7, is obtained from a simple 1:1 stoichiometry model, where the proposed mechanism is not considered. However, the analysis of the data obtained lead to propose an association mechanism such as Scheme 2.

**SCHEME 3:**
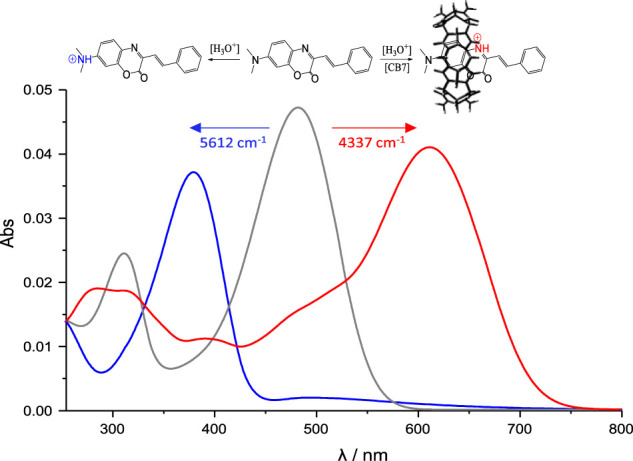
UV-vis spectra of **SAC1** (1.5 µM) at: pH = 2.5 (grey line), pH = 2.5 in presence of 1.2 mM CB7 (red line) and pH = 0 (blue line). All in 30% methanol at 25.0°C.

It is interesting to note that in the proposed mechanism, there is a change of the preferred protonation site in **SAC1-3** in the presence of CB7, which was demonstrated through the optical response associated with the protonation of heterocyclic nitrogen, which until now was unknown. Both observations are summarized for **SAC1** in Scheme 3.

Thus, the protonation would be induced by the CB7 portals and would be responsible for the larger bathochromic effect (≈4500 cm^−1^). Furthermore, the theoretical analysis of the boundary orbitals for **SAC1**, **SAC2** and **SAC3** (Supplementary Table S1) showed that the atom contributing the most to the LUMO orbital is precisely the heterocyclic nitrogen; the LUMO being the unoccupied orbital that contributes the most in the main electronic transition from the HOMO orbital with 71%. This supports the larger optical response observed, associated with the protonation of heterocyclic nitrogen.

## Conclusion

We demonstrated that the synthesis of 7-dialkylamino-aza-coumarin dyes (**SAC1-3**) can be obtained from modifications of the classical methodologies reported.

Protonation in the dialkylamino group in **SAC1** (pKa = 1.30) and **SAC2** (pKa = 2.35) was favored compared to the other basic sites present in the molecule. Interestingly, for the **SAC3** substrate, the protonation took place both in the dialkylamino group (pKa = 1.75) and in the heterocyclic nitrogen (pKa = 1.67) independently and not consecutively. The different protomers of the SACs studied were strongly supported by the theoretical analysis of the corresponding maximum absorption wavelengths.

In the presence of CB7, the large bathochromic effect observed in the studied aza-coumarins, is due to the protonation of heterocyclic nitrogen when interacting with the CB7 portals, forming an inclusion complex with 1:1 stoichiometry.

Considering the optical response of heterocyclic nitrogen of the studied aza-coumarins, greater attention is required towards this point, especially in designing new systems that promote the participation of heterocyclic nitrogen as part of the binding unit, directed towards the development of probes containing this relevant moiety.

## Experimental Section

All solvents and Reagents were purchased from Sigma-Aldrich and used as received.

### Synthesis and Characterization Data

#### Synthesis of the Intermediates 

##### 5-(Dimethylamino)-2-Nitrosophenol (**b**, R_1_ = CH_3_) and 5-(Diethylamino)-2-Nitrosophenol (**b**, R_1_ = CH_2_CH_3_)

These were obtained following the methodology of Hu et al., (Hu et al., 2011). The yields obtained in this case were 86 and 83% for 5-(dimethylamino)-2-nitrosophenol and for 5-(diethylamino)-2-nitrosophenol, respectively.

##### Synthesis of 7-(Dimethylamino)-3-Methyl-2H-benzo[b][1,4]oxazin-2-one (**e**, R_1_ = CH_3_)

To carry this out, modifications were made to the original methodology proposed in the literature (Le Bris, 1985; Hu et al., 2011). For this study, 1.2 g of the previously synthesized compound (**b**, R_1_ = CH_3_) and 0.1 eq of Pd-C were added to 100 ml of ethanol. Subsequently, the solution was treated with H_2_, under vacuum, overnight, and at room temperature. The excess of H_2_ was then extracted from the reaction mixture and replaced with N_2_. Then, the mixture was left to react*in situ* with methyl pyruvate (1.6 eq) under reflux for 3 h. The residue was filtered, and the ethanol was removed under reduced pressure. Then, the solid was purified by flash column chromatography using silica as the stationary phase and petroleum ether/ethyl acetate (3:1) as the eluent. (0.96 g, yield: 65%; yellow solid). ^1^H-NMR (400 MHz, CDCl_3_): δ (ppm) 7.46 (d, *J* = 9.0 Hz, 1H), 6.63 (dd, *J* = 9.1, 2.8 Hz, 1H), 6.38 (d, *J* = 2.8 Hz, 1H), 3.05 (s, 6H), 2.46 (s, 3H). ^13^C-NMR (101 MHz, CDCl_3_): δ (ppm) 154.4; 151.7; 148.6; 147.4; 128.9; 122.7; 109.5; 97.3; 40.3; 20.7.

##### Synthesis of 7-(Diethylamino)-3-Methyl-2H-benzo[b][1,4]oxazin-2-one (**e**, R_1_ = CH_2_CH_3_

This was synthesized from **b** (R_1_ = CH_2_CH_3_), in an analogous way to the previously described methodology. (Yield: 66%; yellow solid). ^1^H-NMR (400 MHz, CDCl_3_): δ (ppm) 7.45 (d, *J* = 9.0 Hz, 1H), 6.61 (dd, *J* = 9.0; 2.5 Hz, 1H), 6.38 (d, *J* = 2.5 Hz, 1H), 3.40 (q, *J* = 7.1 Hz, 4H), 2.45 (s, 3H), 1.20 (t, *J* = 7.1 Hz, 6H). ^13^C-NMR (101 MHz, CDCl_3_): δ (ppm) 154.5, 149.5, 149.1, 146.7, 129.2, 122.3, 109.2, 96.7, 44.9, 20.7, 12.5.

##### Synthesis of (E)-7-(Dimethylamino)-3-Styryl-2H-benzo[b][1,4]oxazin-2-one (**SAC1**)

To carry this out, modifications were made to the original methodology proposed by Bris *et al.*, (Le Bris, 1985). 200 mg of **e** (R_1_ = CH_3_), 0.2 ml of benzaldehyde (2 eq) and 4 ml of 1,4-dioxane were added in a reaction tube. The mixture was brought to 160°C for 39 h, monitored by TLC. Subsequently, 1,4-dioxane was removed under reduced pressure and the residue purified by chromatography with silica column, the first run using dichloromethane and a second one with petroleum ether/ethyl acetate (5:1). (174 mg, yield: 61%; red solid). ^1^H-NMR (400 MHz, CDCl_3_): δ (ppm) 7.94 (d, *J* = 16.3 Hz, 1H), 7.62 (d, *J* = 7.3 Hz, 2H), 7.55 (d, *J* = 9.0 Hz, 1H), 7.42 (d, *J* = 16.3 Hz, 1H), 7.40–7.35 (m, 2H), 7.34–7.29 (m, 1H), 6.68 (dd, *J* = 9.0, 2.7 Hz, 1H), 6.42 (d, *J* = 2.7 Hz, 1H), 3.08 (s, 6H). ^13^C-NMR (101 MHz, CDCl_3_): δ (ppm) 154.1, 151.9, 148.4, 142.5, 136.6, 136.4, 129.7, 128.9, 128.8, 127.6, 123.8, 122.3, 110.1, 97.2, 40.3. IR *υ*_max_: 2904 (NC**-**H), 1715 (C=O), 1618 (HC = CH, *trans*) cm^−1^. ESI-HRMS: m/z calculated for C_18_H_16_N_2_O_2_^.-^, [M]^.-^ = 292.1217; found 292.1219.

##### Synthesis of (E)-7-(Diethylamino)-3-Styryl-2H-benzo[b][1,4]oxazin-2-one (**SAC2**)

100 mg of **e** (R_1_ = CH_2_CH_3_), 0.1 ml of benzaldehyde (2 eq) and 4 ml of 1,4-dioxane were added in a reaction tube. The mixture was brought to 140°C and after 39 h TLC indicated the end of the reaction. Subsequently, the 1,4-dioxane was removed under reduced pressure and the residue purified in the same way as described above for **SAC1**. (59 mg, yield: 43%; solid red). ^1^H-NMR (400 MHz, CDCl_3_): δ (ppm) 7.94 (d, *J* = 16.3 Hz, 1H), 7.62 (d, *J* = 7.3 Hz, 2H), 7.53 (d, *J* = 9.1 Hz, 1H), 7.42 (d, *J* = 16.2 Hz, 1H), 7.40–7.35 (m, 2H), 7.35–7.27 (m, 1H), 6.66 (dd, *J* = 9.1, 2.7 Hz, 1H), 6.42 (d, *J* = 2.7 Hz, 1H), 3.44 (q, *J* = 7.1 Hz, 4H), 1.23 (t, *J* = 7.1 Hz, 6H). ^13^C-NMR (101 MHz, CDCl_3_): δ (ppm) 154.2, 149.9, 148.8, 141.9, 136.7, 136.1, 129.9, 128.8, 128.8, 127.5, 123.5, 122.5, 109.9, 96.7, 45.0, 12.5. IR *υ*_max_: 2963 (NC**-**H), 1718 (C=O), 1620 (HC = CH, *trans*) cm^−1^. ESI-HRMS: m/z calculated for C_20_H_20_N_2_O_2_^.-^, [M]^.-^ = 320,1530; found 320,1531.

##### Synthesis of (E)-3-(2,4-Dihydroxystyryl)-7-(Dimethylamino)-2H-benzo[b][1,4]oxazin-2-one (**SAC3**)

200 mg of **e** (R_1_ = CH_3_), 271 mg of 2,4-dihydroxybenzaldehyde (2 eq) and 4 ml of 1,4-dioxane were added in a reaction tube. The mixture was brought to 150°C for 80 h (monitored by TLC). Then, 1,4-dioxane was removed under reduced pressure and the residue purified by chromatography with silica column, the first one using acetone/hexane (1: 1) and a second chromatography with dichloromethane/ethyl acetate (2: 1). (96 mg, yield: 30%; wine-red solid). ^1^H-NMR (400 MHz, DMSO-*d*_6_): δ (ppm) 9.97 (s, 1H), 9.72 (s, 1H), 7.99 (d, *J* = 16.3 Hz, 1H), 7.48 (d, *J* = 9.0 Hz, 1H), 7.42 (d, *J* = 8.6 Hz, 1H), 7.23 (d, *J* = 16.3 Hz, 1H), 6.73 (dd, *J* = 9.1, 2.7 Hz, 1H), 6.51 (d, *J* = 2.7 Hz, 1H), 6.40 (d, *J* = 2.3 Hz, 1H), 6.31 (dd, *J* = 8.5, 2.4 Hz, 1H), 3.01 (s, 6H). ^13^C-NMR (101 MHz, DMSO-*d*_6_): δ (ppm) 160.3, 158.3, 154.2, 151.7, 148.1, 143.4, 131.9, 129.4, 129.2, 123.6, 117.9, 115.4, 110.5, 108.3, 103.1, 97.5, 40.4. IR *υ*_max_: 3060-3302 (OH, bonded), 2903 (NC**-**H), 1700 (C=O), 1599 (HC = CH, *trans*) cm^−1^. ESI-HRMS: m/z calculated for C_18_H_15_N_2_O_4_^−^, [M-H]^-^ = 323,1037; found 323,1037.

#### Determination of pKa Values for the SAC1-3

1 L of a 30.6% (v/v) methanolic solution was prepared. The solution was divided into two 500 ml solutions. 16.6 ml were extracted from both solutions with the help of a propipette. For the first solution (Solution A) the extracted volume was replaced by Milli-Q water while the second solution (Solution B) was replaced with an HCl solution (11.96 M). Thus, two methanolic solutions were obtained at 29.6% (v/v), one in the absence of HCl and the other at 0.40 M of HCl. Various 2.5 ml solutions of different pH values were prepared by diluting Solution A and Solution B in photometric cells. These small solutions were prepared considering 10 µL of the substrate (375 µM) previously prepared in methanol. The solutions at pH 2.5 to 3.0 were confirmed by an Adwa AD1030 pH-meter. This implied that the HCl concentration of the stock solution was correct, validating the dissolution method used.

The pKa values were determined by the non-linear regression method to fit the UV-vis absorptions and the pH involved in the prepared solutions. The equation used in the adjustment of the experimental data was:

$$\text{A}\text{b}\text{s}=\frac{\left({\text{ε}}_{\text{S}+{\text{H}}^{+}}-{\text{ε}}_{\text{S}}\right)}{2}\left\{\left({\left[\text{S}\right]}_{0}+10^{-\text{p}\text{H}}+10^{-\text{p}\text{K}\text{a}}\right)-\sqrt{{\left({\left[\text{S}\right]}_{0}+10^{-\text{p}\text{H}}+10^{-\text{p}\text{K}\text{a}}\right)}^{2}-4{\left[\text{S}\right]}_{0}10^{-\text{p}\text{H}}}\;\right\}+{\text{ε}}_{\text{S}}{\left[\text{S}\right]}_{0}$$ (1)

where [S]_0_ is the initial substrate concentration, and *ϵ*_S_ and $\varepsilon_{S+H^{+}}$ are the molar absorptivities of the non-protonated and protonated substrate, respectively.

#### Determination of Binding Constants of SAC1-3 in Presence of CB7

**SCHEME 4:**
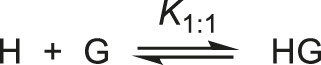
Host-guest binding mechanism for a 1:1 stoichiometric ratio.

An estimated 1250 µM concentration of commercial CB7 (Sigma-Aldrich) was prepared in methanolic solution (3:7 v/v). The exact concentration of the previously prepared solution was determined by titration with cobaltocene hexafluorophosphate, as detailed in the work carried out by Kaifer *et al.* (Yi and Kaifer, 2011). The preparation of the solutions (including the CB7 solution) at different pHs was carried out as described in the previous item. Thus, for a specific pH, different methanolic solutions (3:7 v/v) were prepared at different concentrations of CB7 with 1.5 µM of substrate. Spectrophotometric measurements were performed and the data obtained were adjusted to the binding model of 1:1 stoichiometry (Scheme 4), making use of Eq. 2 (Thordarson, 2011).

$$Abs=\frac{\left(\varepsilon_{HG}-\varepsilon_{G}\right)}{2}\left(–b-\sqrt{b^{2}-4{\left[H\right]}_{0}{\left[G\right]}_{0}}\right)+\varepsilon_{G}{\left[G\right]}_{0};\;with\;b=-\left({\left[H\right]}_{0}+{\left[G\right]}_{0}+\frac{1}{K_{1:1}}\right)$$ (2)

where ${\left[H\right]}_{0}\;$ and ${\left[G\right]}_{0}$ are the initial molar concentrations of the host and guest, and $\;\varepsilon_{G}$ and $\varepsilon_{HG}$ the absorptivities of the guest and complex at the measurement wavelength, respectively.

### Computational Methodology

#### DFT Calculations

The electronic structure calculations for the excited and ground state of the SAC1-3 and complexes (Scheme 1) were carried out using density functional theory (DFT). Two different levels of theory were used: B3LYP-D3/6-31G(d,p) for non- and mono-protonated (on the heterocyclic nitrogen) SACs and ωB97X-D/PBE0/6-31G(d,p) for di- and mono-protonated (on the dialkylamino nitrogen) SACs. The environment (methanol/water) was simulated by use of the solvation model based on density (SMD) (Marenich et al., 2009). All calculations were carried out using the Gaussian 09 package (Frisch et al., 2009). The default parameters for convergence in the optimization routine were used: convergence on the density matrix was 10−9 atomic units, the threshold value for maximum displacement was 0.0018 Å, and the maximum force was 0.00045 Hartree/Bohr.

#### Molecular Dynamic Simulations

CB7 and **SAC3** were prepared using B3LYP-D3/6-31G(d,p) basis set in Gaussian 09 package (Frisch et al., 2009). Macrocycle/SACs Complexes were obtained as described in the literature (Aliaga et al., 2017; Fierro et al., 2021). Subsequently, MD simulations were carried out using the Amber16 suite of programs (Case et al., 2016) and the AMBER ff14SB (Hornak et al., 2006) as force field. Parameters of macrocycles and ligands were obtained using antechamber in the LEAP module of AmberTools.

Each system was solvated in a periodic box using TIP3P explicit water molecules (Mark and Nilsson, 2001). All systems were neutralized by adding counter ions (Na^+^ or Cl^−^) and the molecular dynamic simulations were carried out during 100 ns as described in Momin *et al.,* (Momin et al., 2018). The MM/PBSA approach (Kollman et al., 2000) was used to estimate the binding affinity for each complex.

## Data Availability

The original contributions presented in the study are included in the article/Supplementary Material, further inquiries can be directed to the corresponding authors.

## References

[B1] Anila H. A. Reddy U. G. Ali F. Taye N. Chattopadhyay S. Das A. (2015). A Reagent for Specific Recognition of Cysteine in Aqueous Buffer and in Natural Milk: Imaging Studies, Enzymatic Reaction and Analysis of Whey Protein. Chem. Commun. 51, 15592–15595. 10.1039/c5cc04876a 26355533

[B2] Agarwalla H. Anila H. A. Ali F. Pradhan S. R. Ganguly B. Pramanik S. K. (2018). Fluorescent Chemodosimeter for Quantification of Cystathionine-γ-Synthase Activity in Plant Extracts and Imaging of Endogenous Biothiols. Chem. Commun. 54, 9079–9082. 10.1039/c8cc04296a 30058655

[B3] Agarwalla H. Gangopadhyay M. Sharma D. K. Basu S. K. Jadhav S. Chowdhury A. (2015). Fluorescent Probes for the Detection of Cyanide Ions in Aqueous Medium: Cellular Uptake and Assay for β-glucosidase and Hydroxynitrile Lyase. J. Mater. Chem. B 3, 9148–9156. 10.1039/c5tb01853f 32263129

[B4] Agarwalla H. Pal S. Paul A. Jun Y. W. Bae J. Ahn K. H. (2016). A Fluorescent Probe for Bisulfite Ions: its Application to Two-Photon Tissue Imaging. J. Mater. Chem. B 4, 7888–7894. 10.1039/C6TB02637K 32263779

[B5] Alcázar J. J. Geue N. Valladares V. Cañete A. Pérez E. G. García-Río L. (2021). Supramolecular Control of Reactivity toward Hydrolysis of 7-Diethylaminocoumarin Schiff Bases by Cucurbit[7]uril Encapsulation. ACS Omega 6, 10333–10342. 10.1021/acsomega.1c00683 34056186 PMC8153742

[B6] Aliaga M. E. García-Río L. Numi A. Rodríguez A. Arancibia-Opazo S. Fierro A. (2017). Controlled Keto-Enol Tautomerism of Coumarin Containing β-ketodithioester by its Encapsulation in Cucurbit[7]uril. New J. Chem. 41, 15574–15580. 10.1039/c7nj03265j

[B7] Assaf K. I. Nau W. M. (2015). Cucurbiturils: From Synthesis to High-Affinity Binding and Catalysis. Chem. Soc. Rev. 44, 394–418. 10.1039/c4cs00273c 25317670

[B8] Bagno A. Scorrano G. (2000). Selectivity in Proton Transfer, Hydrogen Bonding, and Solvation. Acc. Chem. Res. 33, 609–616. 10.1021/ar990149j 10995198

[B9] Barooah N. Mohanty J. Pal H. Bhasikuttan A. C. (2012). Stimulus-Responsive Supramolecular pKa Tuning of Cucurbit[7]uril Encapsulated Coumarin 6 Dye. J. Phys. Chem. B 116, 3683–3689. 10.1021/jp212459r 22385336

[B10] Barooah N. Sundararajan M. Mohanty J. Bhasikuttan A. C. (2014). Synergistic Effect of Intramolecular Charge Transfer toward Supramolecular pKa Shift in Cucurbit[7]uril Encapsulated Coumarin Dyes. J. Phys. Chem. B 118, 7136–7146. 10.1021/jp501824p 24881901

[B11] Barrow S. J. Kasera S. Rowland M. J. Del Barrio J. Scherman O. A. (2015). Cucurbituril-Based Molecular Recognition. Chem. Rev. 115, 12320–12406. 10.1021/acs.chemrev.5b00341 26566008

[B12] Basarić N. Thomas S. S. Bregović V. B. Cindro N. Bohne C. (2015). Phototautomerization in Pyrrolylphenylpyridine Terphenyl Systems. J. Org. Chem. 80, 4430–4442. 10.1021/acs.joc.5b00275 25822735

[B13] Basílio N. Gago S. Parola A. J. Pina F. (2017). Contrasting pKa Shifts in Cucurbit[7]uril Host-Guest Complexes Governed by an Interplay of Hydrophobic Effects and Electrostatic Interactions. ACS Omega 2, 70–75. 10.1021/acsomega.6b00427 31457209 PMC6640924

[B14] Behera S. K. Krishnamoorthy G. (2017). Perturbation of Cationic Equilibrium by Cucurbit-7-Uril. Phys. Chem. Chem. Phys. 19, 19234–19242. 10.1039/c7cp03583g 28702607

[B15] Bull J. N. Coughlan N. J. A. Bieske E. J. (2017). Protomer-Specific Photochemistry Investigated Using Ion Mobility Mass Spectrometry. J. Phys. Chem. A. 121, 6021–6027. 10.1021/acs.jpca.7b05800 28723091

[B16] Cao D. Liu Z. Verwilst P. Koo S. Jangjili P. Kim J. S. (2019). Coumarin-Based Small-Molecule Fluorescent Chemosensors. Chem. Rev. 119, 10403–10519. 10.1021/acs.chemrev.9b00145 31314507

[B17] Case D. A. Betz R. M. Cerutti D. S. Cheatham T. E. Darden T. A. III Duke R. E. (2016). AMBER 16. San Francisco: University of California.

[B18] Chen Q. Liu W. Han Y. Li L. Yuan F. Long L. (2020). Accurately Monitoring of Sulfur Dioxide Derivatives in Agricultural Crop Leaf Tissues by a Novel Sensing System. Sensors Actuators B: Chem. 323, 128711. 10.1016/j.snb.2020.128711

[B19] Cox K. A. Gaskell S. J. Morris M. Whiting A. (1996). Role of the Site of Protonation in the Low-Energy Decompositions of Gas-phase Peptide Ions. J. Am. Soc. Mass. Spectrom. 7, 522–531. 10.1016/1044-0305(96)00019-0 24203424

[B20] Demireva M. Armentrout P. B. (2021). Relative Energetics of the Gas Phase Protomers of P-Aminobenzoic Acid and the Effect of Protonation Site on Fragmentation. J. Phys. Chem. A. 125, 2849–2865. 10.1021/acs.jpca.0c11540 33822603

[B21] Depierreux C. Le Bris M. T. Michel M. F. Valeur B. Monsigny M. Delmotte F. (1990). Benzoxazinone-kanamycin Derivative: a New Fluorescent Probe for Flow Cytometry Analysis of Bacteria (Agrobacterium Tumefaciens). FEMS Microbiol. Lett. 67, 237–243. 10.1111/j.1574-6968.1990.tb04026.x

[B22] Dsouza R. N. Pischel U. Nau W. M. (2011). Fluorescent Dyes and Their Supramolecular Host/guest Complexes with Macrocycles in Aqueous Solution. Chem. Rev. 111, 7941–7980. 10.1021/cr200213s 21981343

[B23] Fan J. Mu H. Zhu H. Wang J. Peng X. (2015). Light up ClO−in Live Cells Using an Aza-Coumarin Based Fluorescent Probe with Fast Response and High Sensitivity. Analyst 140, 4594–4598. 10.1039/c5an00777a 25997521

[B24] Fan J. Sun W. Hu M. Cao J. Cheng G. Dong H. (2012). An ICT-Based Ratiometric Probe for Hydrazine and its Application in Live Cells. Chem. Commun. 48, 8117–8119. 10.1039/c2cc34168a 22766565

[B25] Fery-Forgues S. Le Bris M. T. Mialocq J. C. Pouget J. Rettig W. Valeur B. (1992). Photophysical Properties of Styryl Derivatives of Aminobenzoxazinones. J. Phys. Chem. 96, 701–710. 10.1021/j100181a035

[B26] Fierro A. García-Río L. Arancibia-Opazo S. Alcázar J. J. Santos J. G. Aliaga M. E. (2021). Cucurbit[7]uril as a Supramolecular Catalyst in Base-Catalyzed Reactions. Experimental and Theoretical Studies on Carbonate and Thiocarbonate Hydrolysis Reactions. J. Org. Chem. 86, 2023–2027. 10.1021/acs.joc.0c02728 33373222

[B27] Frisch M. J. Trucks G. W. Schlegel H. B. Scuseria G. E. Robb M. A. Cheeseman J. R. (2009). Gaussian 09, Revision D.01. Wallingford CT: Gaussian Inc.

[B28] Fu Y. Finney N. S. (2018). Small-molecule Fluorescent Probes and Their Design. RSC Adv. 8, 29051–29061. 10.1039/c8ra02297f PMC9084556 35547972

[B29] Graton J. Berthelot M. Gal J.-F. Girard S. Laurence C. Lebreton J. (2002). Site of Protonation of Nicotine and Nornicotine in the Gas Phase: Pyridine or Pyrrolidine Nitrogen? J. Am. Chem. Soc. 124, 10552–10562. 10.1021/ja017770a 12197757

[B30] Han S. Yue X. Wang J. Zhang Y. Wang B. Song X. (2020). A Novel Near-Infrared Ratiometric Fluorescent Probe for SO2 Detection with a Large Emission Shift. New J. Chem. 44, 4554–4557. 10.1039/c9nj06343a

[B31] Hornak V. Abel R. Okur A. Strockbine B. Roitberg A. Simmerling C. (2006). Comparison of Multiple Amber Force fields and Development of Improved Protein Backbone Parameters. Proteins 65, 712–725. 10.1002/prot.21123 16981200 PMC4805110

[B32] Hu M. Fan J. Li H. Song K. Wang S. Cheng G. (2011). Fluorescent Chemodosimeter for Cys/Hcy with a Large Absorption Shift and Imaging in Living Cells. Org. Biomol. Chem. 9, 980–983. 10.1039/c0ob00957a 21165468

[B33] Jana P. Mukherjee T. Khurana R. Barooah N. Soppina V. Mohanty J. (2019). Fluorescence Enhancement of Cationic Styrylcoumarin-Cucurbit[7]uril Complexes: Enhanced Stability and Cellular Membrane Localization. J. Photochem. Photobiol. A: Chem. 384, 112062. 10.1016/j.jphotochem.2019.112062

[B34] Kirpichënok M. A. Baukulev V. M. Karandashova L. A. Grandberg I. I. (1991). Synthesis and Spectral and Luminescent Properties of 3-Formyl-7-Dialkylaminocoumarins. Chem. Heterocycl. Compd. 27, 1193–1199. 10.1007/BF00471743

[B35] Kollman P. A. Massova I. Reyes C. Kuhn B. Huo S. Chong L. (2000). Calculating Structures and Free Energies of Complex Molecules: Combining Molecular Mechanics and Continuum Models. Acc. Chem. Res. 33, 889–897. 10.1021/ar000033j 11123888

[B36] Kwon H. Lee K. Kim H.-J. (2011). Coumarin-malonitrile Conjugate as a Fluorescence Turn-On Probe for Biothiols and its Cellular Expression. Chem. Commun. 47, 1773–1775. 10.1039/c0cc04092d 21127785

[B37] Le Bris M.-T. (1985). Synthesis and Properties of Some 7-Dimethylamino-1,4-Benzoxazin-2-Ones. J. Heterocycl. Chem. 22, 1275–1280. 10.1002/jhet.5570220526

[B38] Li M. Feng W. Zhang H. Feng G. (2017). An Aza-Coumarin-Hemicyanine Based Near-Infrared Fluorescent Probe for Rapid, Colorimetric and Ratiometric Detection of Bisulfite in Food and Living Cells. Sensors Actuators B: Chem. 243, 51–58. 10.1016/j.snb.2016.11.132

[B39] Liu X.-D. Sun R. Xu Y. Xu Y.-J. Ge J.-F. Lu J.-M. (2013). A Benzoxazine-Hemicyanine Based Probe for the Colorimetric and Ratiometric Detection of Biothiols. Sensors Actuators B: Chem. 178, 525–531. 10.1016/j.snb.2012.12.108

[B40] Lu Y. Li H. Yao Q. Sun W. Fan J. Du J. (2020). Lysozyme-targeted Ratiometric Fluorescent Probe for SO2 in Living Cells. Dyes and PigmentsPigment 180, 108440. 10.1016/j.dyepig.2020.108440

[B41] Macartney D. H. (2018). Cucurbit[n]uril Host-Guest Complexes of Acids, Photoacids, and Super Photoacids. Isr. J. Chem. 58, 230–243. 10.1002/ijch.201700096

[B42] Manna A. Chakravorti S. (2015). Supramolecular Effect of Curcurbit[7]uril on the Binding Mode of 2-(4-(dimethylamino) Styryl)-1-Methylpyridinium Iodide with Calf Thymus DNA: From Minor Groove to Intercalative. Spectrochimica Acta A: Mol. Biomol. Spectrosc. 150, 120–126. 10.1016/j.saa.2015.05.035 26037496

[B43] Marenich A. V. Cramer C. J. Truhlar D. G. (2009). Universal Solvation Model Based on Solute Electron Density and on a Continuum Model of the Solvent Defined by the Bulk Dielectric Constant and Atomic Surface Tensions. J. Phys. Chem. B 113, 6378–6396. 10.1021/jp810292n 19366259

[B44] Mark P. Nilsson L. (2001). Structure and Dynamics of the TIP3P, SPC, and SPC/E Water Models at 298 K. J. Phys. Chem. A. 105, 9954–9960. 10.1021/jp003020w

[B45] Moghaddam S. Yang C. Rekharsky M. Ko Y. H. Kim K. Inoue Y. (2011). New Ultrahigh Affinity Host−Guest Complexes of Cucurbit[7]uril with Bicyclo[2.2.2]octane and Adamantane Guests: Thermodynamic Analysis and Evaluation of M2 Affinity Calculations. J. Am. Chem. Soc. 133, 3570–3581. 10.1021/ja109904u 21341773 PMC3065999

[B46] Mohanty J. Bhasikuttan A. C. Nau W. M. Pal H. (2006). Host−Guest Complexation of Neutral Red with Macrocyclic Host Molecules: Contrasting pKa Shifts and Binding Affinities for Cucurbit[7]uril and β-Cyclodextrin. J. Phys. Chem. B 110, 5132–5138. 10.1021/jp056411p 16526757

[B47] Momin M. Yao X.-Q. Thor W. Hamelberg D. (2018). Substrate Sequence Determines Catalytic Activities, Domain-Binding Preferences, and Allosteric Mechanisms in Pin1. J. Phys. Chem. B 122, 6521–6527. 10.1021/acs.jpcb.8b03819 29851476

[B48] Monsigny M. Midoux P. Depierreux C. Bebear C. Bris M.-T. Valeur B. (1990). Benzoxazinone Kanamycin A Conjugate. A New Fluorescent Probe Suitable to Detect Mycoplasmas in Cell Culture. Biol. Cel 70, 101–105. 10.1016/0248-4900(90)90365-a 2103517

[B49] Nagarajan R. Varadaraju C. Lee K. H. (2021). Recent Advancements in the Role of N-Heterocyclic Receptors on Heavy Metal Ion Sensing. Dyes Pigm. 191, 109331. 10.1016/j.dyepig.2021.109331

[B50] Patalakha N. S. Yufit D. S. Kirpichenok M. A. Gordeeva N. A. Struchkov Y. T. Grandberg I. I. (1991). Luminescence-spectral and Acid-Base Characteristics of 3-Aryl-7-Diethylaminocoumarins. Chem. Heterocycl. Compd. 27, 32–37. 10.1007/BF00633212

[B51] Paudics A. Hessz D. Bojtár M. Gyarmati B. Szilágyi A. Kállay M. (2020). Binding Modes of a Phenylpyridinium Styryl Fluorescent Dye with Cucurbiturils. Molecules 25, 5111. 10.3390/molecules25215111 PMC7663148 33153219

[B52] Pereira-Vilar A. Martin-Pastor M. Pessêgo M. García-Río L. (2016). Supramolecular Recognition Induces Nonsynchronous Change of Dye Fluorescence Properties. J. Org. Chem. 81, 6587–6595. 10.1021/acs.joc.6b01230 27385129

[B53] Selvan G. T. Poomalai S. Ramasamy S. Selvakumar P. M. Muthu Vijayan Enoch I. V. Lanas S. G. (2018). Differential Metal Ion Sensing by an Antipyrine Derivative in Aqueous and β-Cyclodextrin Media: Selectivity Tuning by β-Cyclodextrin. Anal. Chem. 90, 13607–13615. 10.1021/acs.analchem.8b03810 30412380

[B54] Thordarson P. (2011). Determining Association Constants from Titration Experiments in Supramolecular Chemistry. Chem. Soc. Rev. 40, 1305–1323. 10.1039/c0cs00062k 21125111

[B55] Trebaul C. Roncali J. Garnier F. Guglielmetti R. (1987). Synthesis and Fluorescence Analysis of 3-Substituted 7-Dialkylamino-2h-1,4-Benzoxazin-2-Ones. Bcsj 60, 2657–2662. 10.1246/bcsj.60.2657

[B56] Tu Y.-P. (2006). Dissociative Protonation Sites: Reactive Centers in Protonated Molecules Leading to Fragmentation in Mass Spectrometry. J. Org. Chem. 71, 5482–5488. 10.1021/jo060439v 16839126

[B57] Wu J. Isaacs L. (2009). Cucurbit[7]uril Complexation Drives Thermaltrans-Cis-Azobenzene Isomerization and Enables Colorimetric Amine Detection. Chem. Eur. J. 15, 11675–11680. 10.1002/chem.200901522 19774569

[B58] Wu R. McMahon T. B. (2007). Infrared Multiple Photon Dissociation Spectroscopy as Structural Confirmation for GlyGlyGlyH+ and AlaAlaAlaH+ in the Gas Phase. Evidence for Amide Oxygen as the Protonation Site. J. Am. Chem. Soc. 129, 11312–11313. 10.1021/ja0734492 17718566

[B59] Xu Y. Panzner M. J. Li X. Youngs W. J. Pang Y. (2010). Host-guest Assembly of Squaraine Dye in Cucurbit[8]uril: Its Implication in Fluorescent Probe for Mercury Ions. Chem. Commun. 46, 4073–4075. 10.1039/c002219p 20407667

[B60] Yi S. Kaifer A. E. (2011). Determination of the Purity of Cucurbit[n]uril (N = 7, 8) Host Samples. J. Org. Chem. 76, 10275–10278. 10.1021/jo2018312 22054262

[B61] Yu M. Du W. Li H. Zhang H. Li Z. (2017). Near-infrared Ratiometric Fluorescent Detection of Arginine in Lysosome with a New Hemicyanine Derivative. Biosens. Bioelectron. 92, 385–389. 10.1016/j.bios.2016.10.090 27838202

[B62] Zhang R. Yan X. Guo H. Hu L. Yan C. Wang Y. (2020). Supramolecular Polymer Networks Based on Pillar[5]arene: Synthesis, Characterization and Application in the Fenton Reaction. Chem. Commun. 56, 948–951. 10.1039/c9cc09155f 31854403

[B63] Ziarani G. M. Moradi R. Lashgari N. Kruger H. G. (2018). “Introduction and Importance of Synthetic Organic Dyes,” in Metal-Free Synthetic Organic Dyes (Elsevier), 1–7. 10.1016/b978-0-12-815647-6.00001-7

