# Supplementary material for: Changes in Protonation Sites of 3-Styryl Derivatives of 7-(dialkylamino)-aza-coumarin Dyes Induced by Cucurbit[7]uril
Source: Front Chem. 2022 Apr 14;10:870137. doi: 10.3389/fchem.2022.870137 (PMC9046931; doi:10.3389/fchem.2022.870137)
Supplement: Supplementary file 1 [file DataSheet1.docx]

**Supporting Information (SI)**

**Changes in protonation sites of 3-styryl derivatives of 7-(dialkylamino)-aza-coumarin dyes induced by** **cucurbit[7]uril**

Jackson J. Alcázar,^a^ Edgar Márquez,^b^ Luis García-Río,^c^  Agustín Robles-Muñoz,^a^ Angélica Fierro,^a^ José G. Santos*^,a^ and Margarita E. Aliaga.*^,a^

*^a^ Facultad de Química y de Farmacia, Pontiﬁcia Universidad Católica de Chile, Casilla 306, Santiago 6094411, Chile*

*^b^ Departamento de Química y Biología, Facultad de Ciencias Exactas, Grupo de Investigaciones en Química y Biología, Universidad del Norte, Carrera 51B, Km 5, vía Puerto Colombia, Barranquilla 081007, Colombia*

*^c^ Departamento de Química Física, Centro de Investigación en Química Biológica y Materiales Moleculares (CIQUS), Universidad de Santiago, 15782 Santiago, Spain*

**Content**

[**Figure S1**. ^1^H-RMN spectrum of (E)-7-(dimethylamino)-3-styryl-2H-benzo[b][1,4]oxazin-2-one (SAC1) in CDCl_3_ at 400 MHz and T = 25.0 °C. 2](#_Toc94857957)

[**Figure S2**. ^13^C-RMN spectrum of (*E*)-7-(dimethylamino)-3-styryl-2*H*-benzo[*b*][1,4]oxazin-2-one (**SAC1**) in CDCl_3_ at 101 MHz and T = 25.0 °C. 2](#_Toc94857958)

[**Figure S3**. ^1^H-RMN spectrum of (*E*)-7-(diethylamino)-3-styryl-2*H*-benzo[*b*][1,4]oxazin-2-one (**SAC2**) in CDCl_3_ at 400 MHz and T = 25.0 °C. 3](#_Toc94857959)

[**Figure S4**. ^13^C-RMN spectrum of (*E*)-7-(diethylamino)-3-styryl-2*H*-benzo[*b*][1,4]oxazin-2-one (**SAC2**) in CDCl_3_ at 101 MHz and T = 25.0 °C. 3](#_Toc94857960)

[**Figure S5**. ^1^H-RMN spectrum of (E)-3-(2,4-dihydroxystyryl)-7-(dimethylamino)-2H-benzo[b][1,4]oxazin-2-one (**SAC3**) in DMSO-d*_6_* at 400 MHz and T = 25.0 °C. 4](#_Toc94857961)

[**Figure S6**. ^13^C-RMN spectrum of (*E*)-7-(diethylamino)-3-styryl-2*H*-benzo[*b*][1,4]oxazin-2-one (**SAC3**) in DMSO-d_6_ at 101 MHz and T = 25.0 °C. 4](#_Toc94857962)

[**Table S1**. Photophysical characteristics and involved orbitals for non-, mono- and di-protonated SACs calculated by DFT theory. 5](#_Toc94857963)

[**Figure S7**. A) Hydrogen bond network mediated by water molecules for **SAC3** associated with CB7. B) Hydrogen bond network mediated by water molecules between the positively charged moiety of **SAC3** into CB7. Circle represents coulombic interaction generated by the protonated nitrogen in *N,N*-diethyl ammonium group and the negative electronic density from the carbonyl groups located at the CB7 portal. 6](#_Toc94857964)

[**Table S2.** Photophysical characteristics: free substrates and their complexes, and binding constants 7](#_Toc94857965)

**Figure S8.** UV-vis spectra of **SAC1** (1.5 µM) at different pHs. a) In the absence and b) in the presence of CB7 (1.2 mM). The segmented line in both figures is set at 483 nm…………………………………………………………………………………………...7

[**Figure S9**. ESI-HRMS (Ionization voltage: 3.5 kV and negative polarity) for 1.5 µM of **SAC2** in the presence of 1mM of CB7, dissolved in a methanolic solution at 30% (v/v) and pH 2.5. Scan parameters: Resolution: 140000, AGC target: 1x10^6^, Max. inject time: 200. HESI source: Sheath gas flow: 25, Aux gas flow rate: 3, Sweep gas flow rate: 0, Capillary temp.: 250°C, S-lens RF level: 100, Heater temp: 100°C. 8](#_Toc94857966)

[**Figure S10**. Modification of the absorption spectra for the **SAC2** at pH 0.5 (1.5 μM in 30 % MeOH, T = 25.0 °C) by titration with CB7 (0 – 0.9 mM). 8](#_Toc94857967)

[**Tables S3**. Optimized cartesian coordinates of **SAC1-3** and complexes A-C: 9](#_Toc94857968)

**Figure S1**. ^1^H-RMN spectrum of (E)-7-(dimethylamino)-3-styryl-2H-benzo[b][1,4]oxazin-2-one (SAC1) in CDCl_3_ at 400 MHz and T = 25.0 °C.

# **Figure S2**. ^13^C-RMN spectrum of (*E*)-7-(dimethylamino)-3-styryl-2*H*-benzo[*b*][1,4]oxazin-2-one (**SAC1**) in CDCl_3_ at 101 MHz and T = 25.0 °C.

# **Figure S3**. ^1^H-RMN spectrum of (*E*)-7-(diethylamino)-3-styryl-2*H*-benzo[*b*][1,4]oxazin-2-one (**SAC2**) in CDCl_3_ at 400 MHz and T = 25.0 °C.

# **Figure S4**. ^13^C-RMN spectrum of (*E*)-7-(diethylamino)-3-styryl-2*H*-benzo[*b*][1,4]oxazin-2-one (**SAC2**) in CDCl_3_ at 101 MHz and T = 25.0 °C.

# **Figure S5**. ^1^H-RMN spectrum of (E)-3-(2,4-dihydroxystyryl)-7-(dimethylamino)-2H-benzo[b][1,4]oxazin-2-one (**SAC3**) in DMSO-d*_6_* at 400 MHz and T = 25.0 °C.

# **Figure S6**. ^13^C-RMN spectrum of (*E*)-7-(diethylamino)-3-styryl-2*H*-benzo[*b*][1,4]oxazin-2-one (**SAC3**) in DMSO-d_6_ at 101 MHz and T = 25.0 °C.

# **Table S1**. Photophysical characteristics and involved orbitals for non-, mono- and di-protonated SACs calculated by DFT theory.

| Substrates | λ_max_  (nm) | Molecular orbitals and Main atomic contribution | | Main  configuration |
| --- | --- | --- | --- | --- |
| **SAC1**  (np) | 469 |   HOMO (N7 = 21%) |   LUMO (N4 = 22%) | HOMO→LUMO  (71%)  *f* = 1.265 |
| **SAC1**  (ma) | 383 |   HOMO (C10 = 18%) |   LUMO (N4 = C3 = 19%) | HOMO→LUMO  (71%)  *f* = 1.204 |
| **SAC1**  (mh) | 613 |   HOMO (N7 = 24%) |   LUMO (C3 = 20%, C11 = 17%, N4 = 16%) | HOMO→LUMO  (71%)  *f* = 1.059 |
| **SAC1**  (di) | 437 |   HOMO (C10 = 17%) |   LUMO (C3 = 25%, C11 = 20%) | HOMO→LUMO  (71%)  *f* = 1.363 |
| **SAC2**  (np) | 473 |   HOMO (N7 = 21%) |   LUMO (N4 = 22%) | HOMO→LUMO  (71%)  *f* = 1.317 |
| **SAC2**  (ma) | 384 |   HOMO (C10 = 18%) |   LUMO (N4 = C3 = 19%) | HOMO→LUMO  (71%)  *f* = 1.250 |
| **SAC2**  (mh) | 618 |   HOMO (N7 = 24%) |   LUMO (C3 = 20%, C11= N4 = 16%) | HOMO→LUMO  (71%)  *f* = 1.110 |
| **SAC2**  (di) | 437 |   HOMO (C10 = 17%) |   LUMO (C3 = 25%, C11 = 20%) | HOMO→LUMO  (71%)  *f* = 1.317 |
| **SAC3**  (np) | 480 |   HOMO (N7 = 17%) |   LUMO (N4 = 22%) | HOMO→LUMO  (71%)  *f* = 1.291 |
| **SAC3**  (ma) | 424 |   HOMO (C10 = 18%) |   LUMO (C3 = 19%, N4 = 18%) | HOMO→LUMO  (70%)  *f* = 1.123 |
| **SAC3**  (mh) | 634 |   HOMO (N7 = 22%) |   LUMO (C3 = 20%, C11= 17%, N4 = 14%) | HOMO→LUMO  (71%)  *f* = 1.100 |
| **SAC3**  (di) | 467 |   HOMO (C10 = C12 = 17%) |   LUMO (C3 = 23%, C11 = 19%) | HOMO→LUMO  (70%)  *f* = 1.325 |

(np): non-protonated substrate, (ma) = mono-protonated substrate on the dialkylamine nitrogen (N7), (mh): mono-protonated substrate on the heterocyclic nitrogen (N4) and (di): di-protonated substrate on the dialkylamine and heterocyclic nitrogen. *f* is the oscillator strength.


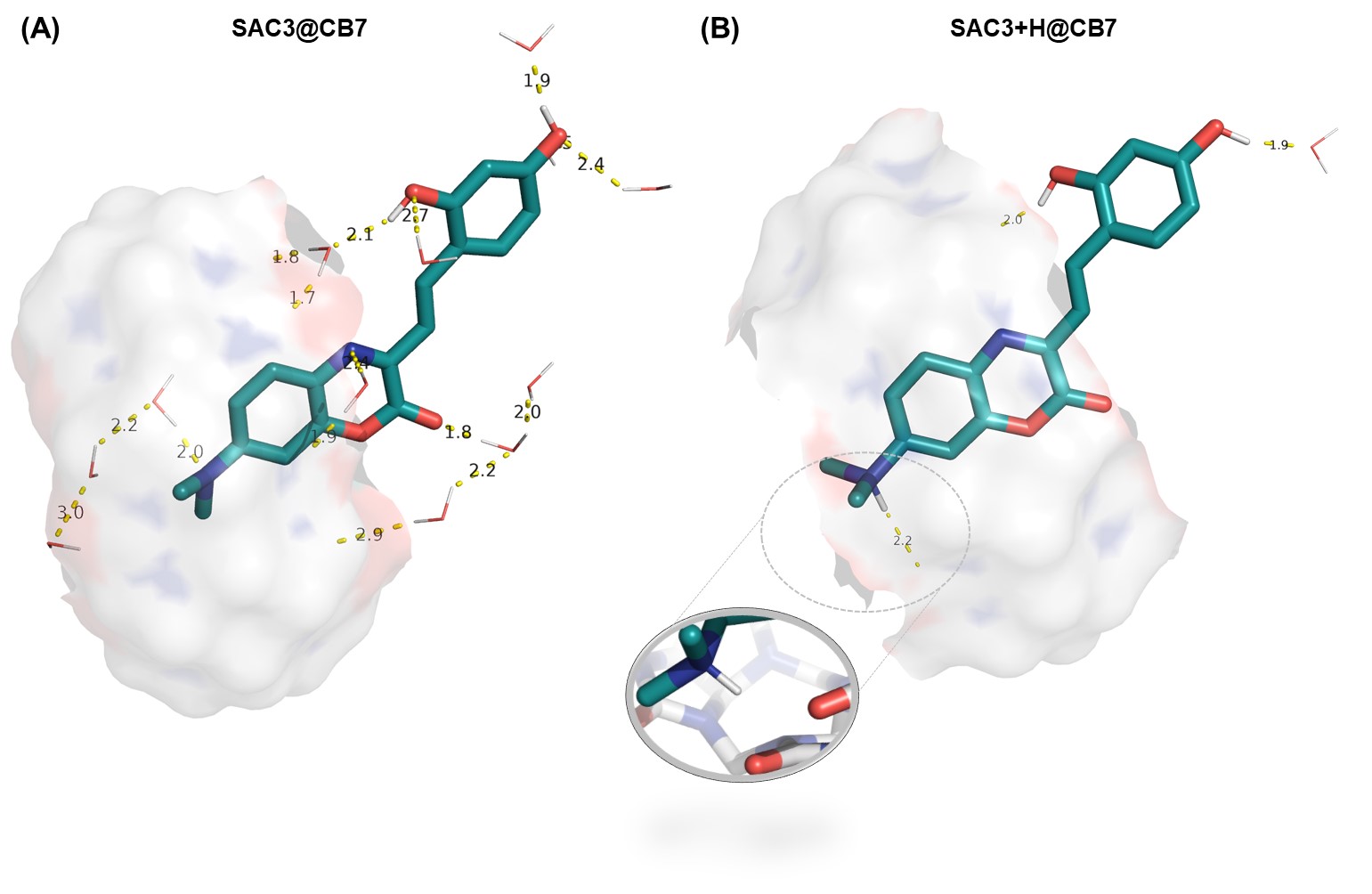


# **Figure S7**. A) Hydrogen bond network mediated by water molecules for non-protonated **SAC3** associated with CB7. B) Hydrogen bond network mediated by water molecules between the positively charged moiety of mono-protonated **SAC3** into CB7. Circle represents coulombic interaction generated by the protonated nitrogen in *N,N*-diethyl ammonium group and the negative electronic density from the carbonyl groups located at the CB7 portal.

Table S2. Photophysical characteristics: free substrates and their complexes, and binding constants (*K*).*

| Species | λ_abs_^H^  (nm) | λ_abs_  (nm) | λ_em_  (nm) | λ_ex_  (nm) | SS  (cm^-1^) | *K* (M^-1^) |
| --- | --- | --- | --- | --- | --- | --- |
| SAC1 | 380^a^ | 483 | 594 | 483 | 3869 |  |
| SAC2 | 380^a^ | 493 | 595 | 493 | 3477 |  |
| SAC3 | 431^a^ | 487 | 621 | 489 | 4431 |  |
| SAC1@CB7^c^ | 611^b^ |  | - | - | - | (3053 ± 159) |
| SAC2@CB7^c^ | 630^b^ |  | - | - | - | (3161 ± 290) |
| SAC3@CB7^c^ | 636^b^ |  | - | - | - | (412 ± 36) |

*MeOH (3:7 v/v) at pH 2.5 and T = 25.0 °C. λ_abs_ and λ_abs_^H^ are the maximum absorption lengths of the neutral and protonated species, respectively. ^a^ Mono-protonated substrate (on the dialkylamine nitrogen). ^b^ Mono-protonated substrate (on the heterocyclic nitrogen) in the complex. ^c^ Complex C. λ_em_ y λ_ex_ are the maximum emission and excitation lengths, respectively. SS is the Stokes shift.

**Figure S8.** UV-vis spectra of **SAC1** (1.5 µM, 30% methanol v/v) at different pHs. a) In the absence of CB7 and b) in the presence of CB7: 1.2 mM for pH 10, 5.3, 2.5 and 0.92 mM for pH 0. The segmented line in both figures is set at 483 nm.

**
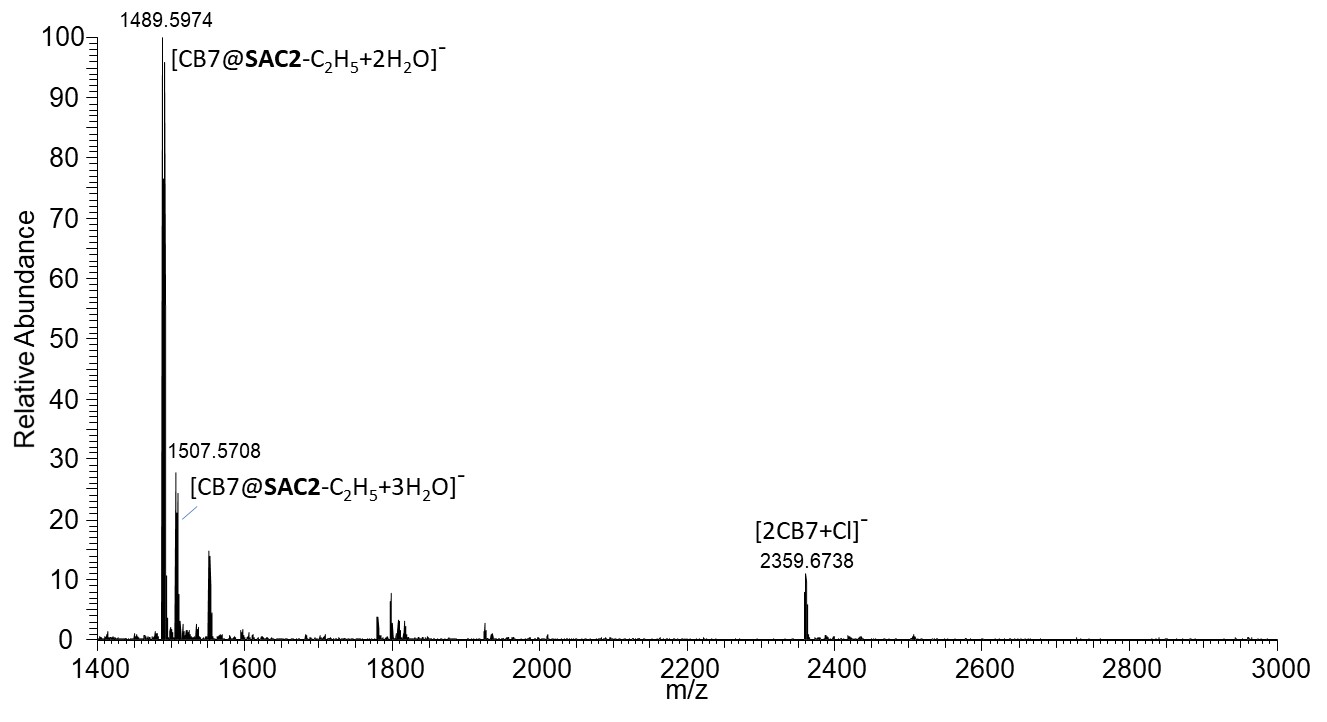
**

# **Figure S9**. ESI-HRMS (Ionization voltage: 3.5 kV and negative polarity) for 1.5 µM of **SAC2** in the presence of 1mM of CB7, dissolved in a methanolic solution at 30% (v/v) and pH 2.5. Scan parameters: Resolution: 140000, AGC target: 1x10^6^, Max. inject time: 200. HESI source: Sheath gas flow: 25, Aux gas flow rate: 3, Sweep gas flow rate: 0, Capillary temp.: 250°C, S-lens RF level: 100, Heater temp: 100°C.

# **Figure S10**. Modification of the absorption spectra for the **SAC2** at pH 0.5 (1.5 μM in 30 % MeOH, T = 25.0 °C) by titration with CB7 (0 – 0.9 mM).

# **Tables S3**. Optimized cartesian coordinates of **SAC1-3** and complexes A-C:

| **SAC1** (non-protonated) | **SAC1** (protonated dimethylamine nitrogen) |
| --- | --- |
| Charge = 0, Multiplicity = 1, Imaginary freq = none)  C 4.40778600 -0.26743600 0.03849300  C 3.47375300 0.80107600 0.07170300  C 2.12162000 0.51888100 0.06562600  C 1.60388900 -0.79349100 0.03333200  C 2.54154700 -1.85084800 0.00311900  C 3.89657700 -1.60570000 0.00429200  H 3.78308700 1.83723500 0.09392600  H 2.16540500 -2.86920900 -0.02121200  H 4.57812700 -2.44563000 -0.01814800  N 5.75033900 -0.02703200 0.03884400  C 6.70287800 -1.13091000 -0.04467400  H 6.54380900 -1.72816400 -0.95024400  H 7.71367600 -0.72628700 -0.07888700  H 6.62921400 -1.79492700 0.82523300  C 6.25053300 1.34270200 0.09720000  H 5.89307000 1.85956200 0.99592000  H 7.33925300 1.32478400 0.12627500  H 5.93965800 1.92348900 -0.78059100  N 0.26212900 -1.03344700 0.02709200  C -0.58921700 -0.02702200 0.04425800  C -0.12268700 1.38101000 0.07279300  O 1.24294700 1.57131500 0.08897500  C -2.00245500 -0.36615200 0.03417900  H -2.15616600 -1.44303800 0.02924200  C -4.47615800 0.05620200 0.00802800  C -4.90211500 -1.28452700 0.12421300  C -5.46355000 1.05366700 -0.12719100  C -6.25586800 -1.60723900 0.09652300  H -4.17203700 -2.07916400 0.24276200  C -6.81925500 0.72863200 -0.15638300  H -5.15606400 2.09293800 -0.21332500  C -7.22237600 -0.60429800 -0.04584100  H -6.56124200 -2.64593700 0.18887000  H -7.56043000 1.51575700 -0.26390800  H -8.27758300 -0.86133700 -0.06632200  O -0.81561400 2.37921200 0.08487600  C -3.07005500 0.46670200 0.02105100  H -2.89593000 1.53696200 0.00829900 | Charge = 1, Multiplicity = 1, Imaginary freq = none  C 4.91063000 1.21483200 0.22367700  C 6.26723600 1.51154700 0.24907000  C 7.21315900 0.50108800 0.07365600  C 6.79239500 -0.81212300 -0.12226800  C 5.43383500 -1.11105300 -0.14357300  C 4.47192700 -0.10348800 0.01986400  C 2.02655100 0.38480700 -0.09415900  C -6.35767400 0.84840900 1.27985300  C -6.47751500 0.68137600 -1.19191800  N -5.75787200 0.24454300 0.04682900  O 0.76347500 -2.31867100 0.02390300  O -1.26254000 -1.46980300 0.05269100  C 0.08902900 -1.32096300 -0.00577400  C 0.59947500 0.08714800 -0.10099300  N -0.20537400 1.10011500 -0.18056300  C -3.81970200 1.76866700 -0.17759500  C -2.45376700 1.97079400 -0.23431100  C -1.57168200 0.88563600 -0.15149800  C -2.09990200 -0.39812600 -0.01891900  C -3.46786400 -0.63177100 0.04474800  C -4.30402500 0.46704900 -0.03445100  H -6.02842200 0.17569900 -2.04529700  H 2.21152100 1.45421600 -0.15464200  H 4.19145000 2.01294200 0.37902000  H -6.26686700 1.93201500 1.22019200  H 7.52269600 -1.60451600 -0.25424300  H -4.49920600 2.61134200 -0.23652200  H -3.84701600 -1.64254000 0.15373600  H -7.52422400 0.39988800 -1.08367500  H 5.10787700 -2.13695000 -0.29316500  H -5.82251400 0.46187800 2.14567500  H -7.40667100 0.55701100 1.31595200  H 8.27277900 0.73656700 0.09725700  H 6.58933900 2.53533000 0.41325400  H -2.04108500 2.96809700 -0.33844200  H -6.38363000 1.76169800 -1.28962200  C 3.05291400 -0.48109700 -0.01752600  H 2.85095200 -1.54645000 0.01241700  H -5.90596900 -0.76715600 0.12251300 |

| **SAC1** (protonated heterocyclic nitrogen) | **SAC1** (di-protonated) |
| --- | --- |
| Charge = 1, Multiplicity = 1, Imaginary freq = none  C -4.29409400 -0.43746600 0.00220700  C -3.52999900 0.76297700 0.01068600  C -2.15674400 0.69102100 -0.01117400  C -1.47036700 -0.53746700 -0.04393100  C -2.21471700 -1.73134600 -0.05615800  C -3.58844600 -1.68630600 -0.03218400  H -3.99178400 1.73995200 0.03525200  H -1.69101900 -2.68193600 -0.08174900  H -4.13622500 -2.61849700 -0.04117400  N -5.64952300 -0.40131900 0.02607200  C -6.42826300 -1.63993100 0.02796500  H -6.19922700 -2.25131700 0.90815000  H -7.48795800 -1.39109100 0.04975400  H -6.23248100 -2.23305100 -0.87232600  C -6.35190000 0.88046200 0.05514700  H -6.11187400 1.48303600 -0.82860800  H -7.42479700 0.69740800 0.06486600  H -6.09120100 1.45577900 0.95127300  N -0.09855600 -0.51043300 -0.05818800  C 0.64688900 0.59488400 -0.03388500  C -0.07128200 1.89747700 -0.01057600  O -1.43989900 1.86514000 -0.00037800  C 2.07299100 0.62726000 -0.02803400  H 2.48149000 1.63009400 -0.05508000  C 4.35033800 -0.42598000 0.01824400  C 5.10401300 0.77071500 -0.00057200  C 5.04254100 -1.65719400 0.03868000  C 6.49255500 0.72758100 -0.00075800  H 4.60383400 1.73357900 -0.01257900  C 6.43396200 -1.69464900 0.03669400  H 4.47435000 -2.58340100 0.05420800  C 7.16335800 -0.50266600 0.01691200  H 7.05909800 1.65389000 -0.01461500  H 6.94943500 -2.65021100 0.05106700  H 8.24916800 -0.52853200 0.01598900  O 0.48260100 2.97254100 0.00049300  C 2.89946200 -0.45619800 0.01602800  H 2.47227500 -1.45720400 0.05741300  H 0.37008000 -1.41672300 -0.08967700 | Charge = 2, Multiplicity = 1, Imaginary freq = none  C 4.95035000 1.23796800 0.05549400  C 6.31420800 1.47675400 0.01239300  C 7.21078400 0.41098100 -0.09590500  C 6.74127200 -0.89945400 -0.15924500  C 5.37574800 -1.14430100 -0.11392100  C 4.46039500 -0.08116500 -0.00773100  C 2.02822100 0.48814800 0.07812700  C -6.50334800 0.79618400 1.15241500  C -6.37086000 0.61776100 -1.31948500  N -5.76321900 0.21017900 -0.01139500  O 0.79938800 -2.23137500 0.15533100  O -1.23138000 -1.39724800 0.10887500  C 0.11137800 -1.24976300 0.12076000  C 0.65022800 0.16096300 0.09105200  N -0.23829900 1.13658700 0.08203300  C -3.86185800 1.79482800 0.02445800  C -2.50114700 2.03975200 0.04667000  C -1.61815400 0.96106500 0.07428100  C -2.09769000 -0.34109300 0.08337900  C -3.45782300 -0.60666900 0.06421100  C -4.31790000 0.47645100 0.03371400  H -5.82279000 0.12202500 -2.11935600  H 2.23690500 1.55382800 0.08947400  H 4.26840500 2.07716900 0.13987100  H -6.48519700 1.88178300 1.07070300  H 7.43972800 -1.72542200 -0.24233400  H -4.55824100 2.62503300 -0.00459400  H -3.81210000 -1.63199100 0.06872500  H -7.41306700 0.30147400 -1.31281800  H 5.00192900 -2.16283200 -0.16326900  H -6.01822400 0.46464900 2.06900500  H -7.52830000 0.43069500 1.10464800  H 8.27812200 0.60578700 -0.13087100  H 6.68595300 2.49473200 0.06371000  H -2.11499200 3.05293900 0.03795000  H -6.30118400 1.70043000 -1.41386300  C 3.05156600 -0.41171900 0.02688400  H 2.81670600 -1.47012800 0.00094500  H -5.88418500 -0.80553200 0.05776300  H 0.10663400 2.09713600 0.06761900 |

| **SAC2** (non-protonated) | **SAC2** (protonated dimethylamine nitrogen) |
| --- | --- |
| Charge = 0, Multiplicity = 1, Imaginary freq = none)  C -3.76514800 -0.28292600 -0.06239500  C -2.82557400 0.77143400 0.09117200  C -1.47403000 0.49358800 0.04353200  C -0.95443900 -0.80378000 -0.15475800  C -1.89488200 -1.84779800 -0.31123900  C -3.24908400 -1.60567000 -0.26783100  H -3.13027900 1.80035500 0.22609400  H -1.52116100 -2.85673400 -0.45865300  H -3.92734700 -2.44178700 -0.37228100  N -5.10902200 -0.04771300 -0.01376500  C -6.09795300 -1.09658200 -0.29091900  H -6.98585300 -0.60093700 -0.69388700  H -5.72734500 -1.74887800 -1.08601400  C -5.65543400 1.27350500 0.31740300  H -6.62460600 1.10932900 0.79735700  H -5.02049900 1.75228800 1.06779100  N 0.38622300 -1.04091400 -0.18645600  C 1.23699300 -0.04289200 -0.04419500  C 0.76939800 1.35207400 0.14068200  O -0.59672400 1.53755600 0.18605700  C 2.65056800 -0.37912600 -0.07743300  H 2.80479800 -1.44695600 -0.21647200  C 5.12444700 0.04084600 0.01241500  C 5.55366400 -1.29459200 -0.14609400  C 6.11082000 1.03858900 0.15379600  C 6.90907800 -1.61097200 -0.16034100  H 4.82471400 -2.09125800 -0.25691400  C 7.46829300 0.72014000 0.13930100  H 5.80111900 2.07351000 0.27628800  C 7.87462700 -0.60717500 -0.01714100  H 7.21647700 -2.64601900 -0.28236000  H 8.20829200 1.50792700 0.25014100  H 8.93109700 -0.85954700 -0.02797000  O 1.46082200 2.34477800 0.25715500  C 3.71721800 0.44633100 0.04396500  H 3.54115600 1.50760000 0.18052100  C -6.47211400 -1.90825900 0.95104200  H -7.22853900 -2.65882700 0.69759200  H -5.59986800 -2.42431500 1.36382500  H -6.88369500 -1.25738300 1.72967500  C -5.82437000 2.17451400 -0.90798900  H -6.25066800 3.13910400 -0.61203300  H -4.86353000 2.35944700 -1.39823000  H -6.49696600 1.71290300 -1.63859600 | Charge = 1, Multiplicity = 1, Imaginary freq = none  C -3.77413800 0.14029600 -0.02878300  C -2.83899900 1.15514700 -0.14016800  C -1.49763000 0.79924000 -0.12798500  C -1.08381700 -0.52726800 -0.00692600  C -2.05914900 -1.52547400 0.10565000  C -3.40373800 -1.20043000 0.09461400  H -3.12830800 2.19725200 -0.23055900  H -1.73851400 -2.55664400 0.20389900  H -4.14864600 -1.98108200 0.18727000  N -5.19340600 0.52651400 -0.06919300  C -6.05460800 -0.11302900 0.99425300  H -7.05485900 0.28943200 0.82731000  H -6.07912000 -1.18587600 0.80921100  C -5.79246800 0.42751600 -1.46372600  H -6.77230600 0.90096500 -1.38087900  H -5.15500100 1.04882200 -2.09489500  N 0.25920700 -0.86079700 0.01469900  C 1.14870000 0.07835200 -0.07442600  C 0.76234800 1.52276800 -0.21240300  O -0.57078200 1.79149800 -0.23178300  C 2.54637200 -0.33404200 -0.02878700  H 2.64445900 -1.41210100 0.06893200  C 5.02899500 -0.01113600 -0.01716000  C 5.39193100 -1.35479600 0.17029300  C 6.04691300 0.94510600 -0.14766500  C 6.73026400 -1.72350600 0.21977700  H 4.62936300 -2.11919600 0.28162200  C 7.38703400 0.57483100 -0.09843000  H 5.77986200 1.98884700 -0.28983700  C 7.73229700 -0.76173400 0.08449900  H 6.99388200 -2.76647200 0.36626900  H 8.16009000 1.33011800 -0.20090400  H 8.77707900 -1.05426600 0.12532500  O 1.52085400 2.45385300 -0.30882100  C 3.63713500 0.45172300 -0.08296400  H 3.50942700 1.52363600 -0.18724300  C -5.55253200 0.21610400 2.38547900  H -6.27042900 -0.17534400 3.11027000  H -4.58160700 -0.24193000 2.58830700  H -5.47345800 1.29738000 2.53317700  C -5.90185000 -0.98132000 -2.00526100  H -6.37227600 -0.91180500 -2.98943900  H -4.92426200 -1.45005700 -2.13561300  H -6.52994900 -1.62043700 -1.38042300  H -5.21857300 1.52937700 0.14265100 |

| **SAC2** (protonated heterocyclic nitrogen) | **SAC2** (di-protonated) |
| --- | --- |
| Charge = 1, Multiplicity = 1, Imaginary freq = none  C -3.66437400 -0.35714800 -0.05710900  C -2.88324600 0.82418900 0.09624600  C -1.51141000 0.74225400 0.06824000  C -0.83545300 -0.47983800 -0.10889300  C -1.59287000 -1.65451900 -0.27213600  C -2.96541300 -1.59762300 -0.24973500  H -3.33151900 1.80023900 0.21748800  H -1.08135900 -2.60278800 -0.40575200  H -3.51714700 -2.52066800 -0.36008400  N -5.01998000 -0.31272400 -0.01823800  C -5.85410000 -1.49344600 -0.28938600  H -6.79652100 -1.12556500 -0.70392200  H -5.38908700 -2.09577500 -1.07282900  C -5.75116400 0.92082300 0.30714800  H -6.68679700 0.61807600 0.78474200  H -5.19234300 1.48713000 1.05630300  N 0.53487200 -0.46494700 -0.10910100  C 1.29162500 0.62447100 0.03385400  C 0.58629300 1.92333000 0.18867200  O -0.78326900 1.90094800 0.20915000  C 2.71840900 0.63845400 0.04920700  H 3.13798800 1.63007800 0.16763200  C 4.98261400 -0.44454800 -0.04311700  C 5.75604300 0.72981600 0.10676500  C 5.65402300 -1.68026900 -0.17562200  C 7.14366200 0.66087100 0.12328800  H 5.27162500 1.69516100 0.21098800  C 7.04441700 -1.74360400 -0.15892200  H 5.07081800 -2.58997500 -0.29054200  C 7.79376900 -0.57352700 -0.00874400  H 7.72585300 1.57024300 0.23918200  H 7.54338100 -2.70255800 -0.26175200  H 8.87877500 -0.61949700 0.00549600  O 1.14927900 2.98804100 0.30079700  C 3.53131300 -0.44996700 -0.06418800  H 3.09156000 -1.43956900 -0.17877800  C -6.12097200 -2.32937700 0.96342000  H -6.77373600 -3.17308700 0.71631000  H -5.18973200 -2.72373600 1.38133500  H -6.61521400 -1.72774700 1.73311800  C -6.04216500 1.77814500 -0.92565700  H -6.60749400 2.66973700 -0.63489900  H -5.11602100 2.10095000 -1.41110900  H -6.63556600 1.21857000 -1.65599500  H 0.99421600 -1.36835300 -0.22513300 | Charge = 2, Multiplicity = 1, Imaginary freq = none  C -3.72557800 0.01456800 0.04217000  C -2.78770500 1.03422300 0.04286100  C -1.44975200 0.68216400 0.00184600  C -1.05727300 -0.64861100 -0.03813900  C -2.01221300 -1.66330000 -0.04068800  C -3.35501000 -1.33025300 -0.00117700  H -3.07352300 2.08046700 0.07622100  H -1.69622900 -2.70023200 -0.07139300  H -4.09695200 -2.11890900 0.00051800  N -5.14193100 0.41010100 0.05960300  C -6.00867700 -0.36689100 1.02360900  H -7.01104200 0.04432100 0.89566100  H -6.02123200 -1.40760300 0.70461900  C -5.73649000 0.51112300 -1.33848500  H -6.70391700 0.99494100 -1.19501000  H -5.07906200 1.19248100 -1.88067300  N 0.30778100 -0.91426300 -0.06443100  C 1.25798500 0.00146800 -0.06366400  C 0.81228700 1.44511100 -0.05471400  O -0.51616100 1.67989600 0.00407200  C 2.61102000 -0.41637400 -0.06536500  H 2.74632700 -1.49366300 -0.08138500  C 5.07592200 -0.00533800 -0.00574400  C 5.47850600 -1.35442000 0.04134900  C 6.05980000 1.00042700 -0.01938700  C 6.82534700 -1.67742000 0.07366000  H 4.74224600 -2.15109300 0.06120300  C 7.40777100 0.67111700 0.00990200  H 5.75257600 2.04156800 -0.05447300  C 7.79085800 -0.66774600 0.05717500  H 7.13009200 -2.71788400 0.11367300  H 8.15883100 1.45379200 -0.00318400  H 8.84399100 -0.92925000 0.08491000  O 1.56195100 2.38030500 -0.09525100  C 3.69146200 0.41481100 -0.03508500  H 3.52706800 1.48657500 -0.02904100  C -5.52355700 -0.20968500 2.45016900  H -6.23149100 -0.71691600 3.10982600  H -4.53798600 -0.65690300 2.60034000  H -5.48424700 0.84475200 2.73984700  C -5.87822500 -0.81339100 -2.05613200  H -6.33217600 -0.60701900 -3.02876700  H -4.91356200 -1.29344500 -2.23552200  H -6.53434900 -1.50652600 -1.52503000  H -5.16109200 1.37364000 0.41147100  H 0.58959000 -1.89505400 -0.07556600 |

| **SAC3** (non-protonated) | **SAC3** (protonated dimethylamine nitrogen) |
| --- | --- |
| Charge = 0, Multiplicity = 1, Imaginary freq = none  C -5.03316800 -0.26188200 0.00030900  C -4.11298300 0.81827100 -0.00003700  C -2.75628500 0.55250600 -0.00021000  C -2.22123800 -0.75160300 -0.00014400  C -3.14505400 -1.82017900 0.00010400  C -4.50428400 -1.59247400 0.00031100  H -4.43501600 1.85094000 -0.00010600  H -2.75647600 -2.83423100 0.00011400  H -5.17405000 -2.44207800 0.00044600  N -6.38101300 -0.03981600 0.00064800  C -7.31510100 -1.16161500 0.00082900  H -7.18707700 -1.79119200 0.88979800  H -8.33423900 -0.77689100 0.00196000  H -7.18861800 -1.79032800 -0.88899800  C -6.89974300 1.32352500 -0.00007900  H -6.57347500 1.87752000 -0.88931900  H -7.98880500 1.29345000 0.00020000  H -6.57305000 1.87858900 0.88833000  N -0.87447600 -0.97553800 -0.00026900  C -0.03370600 0.04049200 -0.00042600  C -0.52279000 1.44369700 -0.00054600  O -1.89018400 1.61670200 -0.00044700  C 1.38209500 -0.28655800 -0.00045700  H 1.55515400 -1.35558400 -0.00054800  C 3.86098300 0.27039200 -0.00005500  C 4.44849100 -1.02547400 -0.00042200  C 4.75768200 1.36193900 0.00058900  C 5.83343600 -1.19265000 -0.00011500  C 6.13733400 1.21402900 0.00088300  H 4.33954200 2.36481200 0.00087200  C 6.67923300 -0.07945800 0.00053600  H 6.26382200 -2.19052600 -0.00036900  H 6.79182600 2.08131500 0.00138100  O 0.15491500 2.45289400 -0.00073300  C 2.43586600 0.57153100 -0.00023800  H 2.21533000 1.63359600 -0.00010800  O 3.62323200 -2.10715700 -0.00103500  H 4.15262300 -2.92173800 -0.00131800  O 8.01722900 -0.31807700 0.00080200  H 8.49476600 0.52800200 0.00130300 | Charge = 1, Multiplicity = 1, Imaginary freq = none  C 4.48491900 0.98695800 -0.03205600  C 5.87260400 1.09865200 -0.01193500  C 6.66775600 -0.04442500 0.05256100  C 6.07729200 -1.31239400 0.10030100  C 4.69772300 -1.40394200 0.08029600  C 3.85431600 -0.27972800 0.01428000  C 1.40689500 0.36839300 -0.05810100  C -6.98382200 0.65872800 1.33219000  C -7.10799700 0.79201500 -1.14100000  N -6.38203500 0.21437200 0.03448400  O 0.13328800 -2.34251100 -0.03822700  O -1.88695400 -1.48915600 -0.02908300  C -0.53476300 -1.33931100 -0.03978500  C -0.01771200 0.07236400 -0.05611000  N -0.82601700 1.08904700 -0.07152400  C -4.44279900 1.75710800 -0.03646900  C -3.07567900 1.96240400 -0.06165900  C -2.19232900 0.87445200 -0.05907200  C -2.72362600 -0.41437600 -0.03462700  C -4.09161100 -0.65233700 -0.01248700  C -4.92879900 0.44874000 -0.01343400  H -6.63471300 0.42430800 -2.04997900  H 1.60715900 1.43172100 -0.10088100  H -6.88683900 1.74072700 1.40964200  H 6.69367800 -2.20511400 0.15185000  H -5.12137900 2.60278600 -0.03089900  H -4.47038900 -1.66887200 0.00936600  H -8.14372600 0.45911900 -1.08435600  H 4.23682000 -2.38684800 0.11809800  H -6.45119200 0.16349300 2.14283500  H -8.03410300 0.36978100 1.32897900  H 6.34540900 2.07556500 -0.04925900  H -2.66331600 2.96508800 -0.07966000  H -7.05756500 1.87837400 -1.08741800  C 2.41885400 -0.52527800 0.00044100  H 2.15974800 -1.57773700 0.04623400  H -6.52489500 -0.79963200 -0.01480500  O 3.70830500 2.08984800 -0.09723300  H 4.26392200 2.88085200 -0.12514500  O 8.00501800 0.13579000 0.06659500  H 8.44574800 -0.72395400 0.11000700 |

| **SAC3** (protonated heterocyclic nitrogen) | **SAC3** (di-protonated) |
| --- | --- |
| Charge = 1, Multiplicity = 1, Imaginary freq = none  C 4.90958300 -0.49690300 0.00049000  C 4.15703800 0.70581700 -0.08297900  C 2.78062300 0.64850900 -0.05634700  C 2.08184400 -0.56469100 0.04478400  C 2.81416300 -1.76028100 0.12920000  C 4.19154100 -1.73099800 0.10877100  H 4.62704500 1.67665200 -0.15774500  H 2.28406400 -2.70447900 0.20947200  H 4.72676600 -2.66840300 0.17505200  N 6.26997600 -0.47418900 -0.01980700  C 7.03573400 -1.71184400 0.11074600  H 6.81642700 -2.40674500 -0.70827100  H 8.09841900 -1.47609200 0.08104500  H 6.82164700 -2.21602200 1.06064400  C 6.98046400 0.79730800 -0.12527100  H 6.76141400 1.44976900 0.72901700  H 8.05235900 0.60659600 -0.14513800  H 6.70853300 1.32840600 -1.04507500  N 0.70447900 -0.52170200 0.06744100  C -0.02865300 0.59417500 0.01092700  C 0.71297300 1.88608400 -0.06993500  O 2.07804600 1.83257400 -0.12233500  C -1.44525500 0.65901500 0.02031200  H -1.84356900 1.66174400 0.03792800  C -3.71953100 -0.45547700 -0.01194800  C -4.57574300 0.68275000 0.12619900  C -4.35552300 -1.71801400 -0.15743000  C -5.95941400 0.54270300 0.10799900  C -5.72637800 -1.86749100 -0.17935500  H -3.72701000 -2.59788000 -0.26026900  C -6.53664100 -0.72227200 -0.04538000  H -6.60164300 1.41180500 0.21325300  H -6.18197100 -2.84623300 -0.29684000  O 0.17582000 2.96935200 -0.09978800  C -2.28720700 -0.42691200 -0.01761900  H -1.84480500 -1.42039800 -0.08088200  O -4.00214500 1.89734600 0.28201200  H -4.68952000 2.57980700 0.37088000  O -7.88422300 -0.78120200 -0.05517900  H -8.17418300 -1.70205000 -0.17495800  H 0.22716000 -1.41866800 0.14179300 | Charge = 2, Multiplicity = 1, Imaginary freq = none  C 4.48334200 0.98508400 -0.04161200  C 5.86738100 1.08032100 -0.02773500  C 6.64502300 -0.07611100 0.04041700  C 6.04493400 -1.34998800 0.09903800  C 4.67674900 -1.43332700 0.08494600  C 3.83869800 -0.28784900 0.01587700  C 1.41257000 0.40640700 -0.04415400  C -6.99164100 0.57925200 1.32678900  C -7.11384400 0.73013300 -1.14679000  N -6.37841100 0.15750300 0.02622300  O 0.17154100 -2.31152600 -0.05258500  O -1.84797100 -1.46068400 -0.04422100  C -0.50431700 -1.31943800 -0.04678900  C 0.04740400 0.08678900 -0.04696900  N -0.84683500 1.07289000 -0.05787000  C -4.47181700 1.73755000 -0.02462200  C -3.10933500 1.97853700 -0.04435300  C -2.22509800 0.90031100 -0.05052500  C -2.71141100 -0.39933600 -0.04073700  C -4.07165200 -0.66220200 -0.02458000  C -4.93202800 0.42170800 -0.01593700  H -6.62879500 0.38331900 -2.05774700  H 1.63934200 1.46534600 -0.07256400  H -6.91942600 1.66264400 1.41083600  H 6.66050400 -2.24237200 0.15337000  H -5.16562800 2.57045200 -0.01199500  H -4.42702800 -1.68718200 -0.01415000  H -8.14092700 0.37081900 -1.09635400  H 4.20336500 -2.40908400 0.12945600  H -6.44877800 0.09037600 2.13442300  H -8.03477200 0.26587900 1.31899100  H 6.35526900 2.04853300 -0.07353500  H -2.72280600 2.99164400 -0.05100900  H -7.09016200 1.81677700 -1.08093800  C 2.43798200 -0.51743400 0.00614700  H 2.15952600 -1.56437500 0.04805100  H -6.49953000 -0.85877700 -0.03192200  O 3.71444300 2.07987400 -0.11130300  H 4.26419100 2.87665200 -0.14855300  O 7.97184200 0.08539100 0.04761700  H 8.41199400 -0.77636500 0.09427800  H -0.49753700 2.02962800 -0.06076700 |

| **Complex A** (R_1_=CH_3_ and R_2_=H) | **Complex** **B** (R_1_=CH_3_ and R_2_=H) | **Complex C** (R_1_=CH_3_ and R_2_=H) |
| --- | --- | --- |
| Charge = 1, Multiplicity = 1,  Imaginary freq = none  O 4.25810900 0.74539600 3.75801700  C 4.79706100 0.85113000 2.67131700  N 5.40095400 -0.16480500 1.95739000  C 5.56983700 -1.49213900 2.50945300  N 4.77334000 -2.51325700 1.87255000  C 5.13850800 -3.18330500 0.65106200  N 4.99204000 -2.39513600 -0.55493300  C 4.10927300 -2.95326100 -1.44279900  O 3.87358200 -2.53632600 -2.57152400  N 3.57338500 -4.07233000 -0.86543000  C 2.78034600 -5.02708400 -1.59534800  N 1.36734100 -4.93268400 -1.34847200  C 0.47287800 -4.63175000 -2.34737000  O 0.75401700 -4.25781200 -3.47644700  N -0.78992100 -4.87694700 -1.86735200  C -1.95473800 -4.86293000 -2.71552900  N -2.88294900 -3.80147800 -2.43041800  C -3.00275800 -2.68534700 -3.22550600  O -2.30829400 -2.41053900 -4.18995500  N -4.06970000 -1.95126900 -2.75370700  C -4.60641700 -0.83902900 -3.49380600  N -4.48825800 0.42207700 -2.80325800  C -3.68300200 1.43181000 -3.28995200  O -2.84588200 1.31928400 -4.17037100  N -4.01393500 2.58902000 -2.62846200  C -3.44015200 3.86439900 -2.97232700  N -2.39689600 4.30478200 -2.07841700  C -1.11547800 4.55064000 -2.52731500  O -0.66268000 4.24360900 -3.61669900  N -0.45550500 5.24778000 -1.53976100  C 0.84666300 5.83204800 -1.73367900  N 1.91418600 5.18141600 -1.01797700  C 2.80810200 4.33664800 -1.62803700  O 2.79791800 4.00444700 -2.80374400  N 3.74597100 3.96945200 -0.69056500  C 3.59629900 4.70398100 0.55075800  N 3.42399200 3.88841000 1.72290600  C 4.51469400 3.29729800 2.45970500  N 4.95899700 2.01967100 1.95897100  C 5.82629900 1.86053700 0.81763400  N 5.18557100 2.04281600 -0.46966600  C 4.97915500 3.33530500 -1.07399300  C 5.13700100 0.88145500 -1.19515600  O 4.67603700 0.75051700 -2.32532200  N 5.73037900 -0.10530000 -0.45880400  C 6.16896500 0.34114200 0.83939300  C 5.96430600 -1.42183100 -0.97816100  C 2.12113100 3.87790900 2.16815500  O 1.70721700 3.31482100 3.16610600  N 1.37326900 4.66020400 1.30768700  C 2.21399700 5.39017300 0.37550300  C 0.06740400 5.13683700 1.69657900  N -0.98930200 4.76164700 0.78919200  C -1.29248100 5.52956400 -0.40228600  C -2.68100900 4.98630600 -0.83251300  N -3.06077000 4.13318400 0.26384800  C -2.07590200 4.02608900 1.21939700  O -2.16207000 3.42491100 2.27648500  C -4.39417600 3.62518400 0.45364500  N -4.67453500 2.41166700 -0.27827100  C -5.07599100 2.40105200 -1.67341200  C -5.50670900 0.92398900 -1.90203700  N -5.49682000 0.36607800 -0.57294600  C -5.05100200 1.25812200 0.37168800  O -5.03573000 1.07853400 1.57985500  C -5.95672900 -0.95872900 -0.23849500  N -4.99556600 -2.00413400 -0.48693500  C -4.82770800 -2.67279900 -1.75036400  C -3.89036300 -3.86227600 -1.40559100  N -3.43839200 -3.53993400 -0.06236500  C -4.23377900 -2.56723200 0.51158000  O -4.28278500 -2.28691500 1.69646500  C -2.73163500 -4.48803900 0.76572600  N -1.31724800 -4.56623000 0.49219100  C -0.78027100 -5.43143400 -0.53372400  C 0.73231400 -5.50831200 -0.18547100  N 0.84587800 -4.71988200 1.01830400  C -0.36626000 -4.20378600 1.42363200  O -0.56808300 -3.56533200 2.44416100  C 1.99151900 -4.79110400 1.89430900  N 3.08194500 -3.92195000 1.51979900  C 4.03725700 -4.26859400 0.48620400  C 3.60042400 -2.99851100 2.40912500  O 3.12854100 -2.70308400 3.49141000  H 5.26297500 -1.44569300 3.55506700  H 6.62438700 -1.77552000 2.43462700  H 6.15591400 -3.57988100 0.72098500  H 3.12718500 -6.03045700 -1.32627300  H 2.93494700 -4.84832000 -2.65960100  H -1.60564000 -4.72930500 -3.73967300  H -2.47901800 -5.81889100 -2.61415400  H -4.03835700 -0.76248300 -4.42159700  H -5.66422700 -1.02580900 -3.71252700  H -4.24257100 4.61030700 -2.98414500  H -2.99574000 3.77037800 -3.96355600  H 0.80917600 6.87973100 -1.41953900  H 1.07879500 5.76184000 -2.79659700  H 4.43254000 5.39719800 0.68606300  H 5.35468500 3.99931800 2.44480600  H 4.17641000 3.13575100 3.48375200  H 6.69581800 2.51961700 0.89992500  H 4.94861300 3.20188100 -2.15589900  H 5.82108000 3.98017300 -0.79700900  H 7.23159200 0.11946900 0.97546100  H 6.95651400 -1.75034000 -0.65273900  H 5.92515300 -1.36251200 -2.06582800  H 2.20507100 6.45873600 0.61239600  H -0.16384400 4.69848300 2.66811000  H 0.09644900 6.23109300 1.77359500  H -1.28094900 6.60147500 -0.17864200  H -3.43983000 5.76009800 -0.98977900  H -4.51575900 3.39293100 1.51222300  H -5.10444900 4.40269000 0.15095300  H -5.87099200 3.13461100 -1.84196500  H -6.49868500 0.81125900 -2.35039100  H -6.86089000 -1.17239100 -0.81578900  H -6.17866900 -0.96244400 0.82919400  H -5.80048600 -2.97184800 -2.15410500  H -4.38475000 -4.83943100 -1.41576300  H -3.16571900 -5.48585300 0.62713600  H -2.85748300 -4.16380400 1.79941500  H -1.29458600 -6.39798200 -0.52284600  H 1.10489900 -6.52270800 -0.01064600  H 1.66987200 -4.48695800 2.89086100  H 2.34428100 -5.82909800 1.91107900  H 4.39100100 -5.29592000 0.61829800  C -3.09165600 -0.65612900 6.53527700  C -4.11987500 -0.66677000 7.47266400  C -5.41426000 -0.32508400 7.08875400  C -5.66994800 0.02934600 5.76352300  C -4.64530800 0.03788800 4.82468500  C -3.33778400 -0.31634700 5.19713700  C -2.21494000 -0.35203900 4.24969300  C -2.34518100 -0.27136700 2.91445700  C -1.29156300 -0.33366100 1.91026300  N -1.66798600 -0.32868500 0.67178800  C -0.70993800 -0.37025400 -0.32393800  C -1.10835200 -0.34168700 -1.66258300  C 0.65173600 -0.42425400 -0.02781300  C -0.16962400 -0.34862900 -2.67557800  C 1.18499000 -0.39926600 -2.34121400  C 1.62006000 -0.44394900 -1.02573900  O 1.05817800 -0.43639400 1.27258900  C 0.15618300 -0.37613500 2.29550600  O 0.57566100 -0.33747900 3.42101100  N 2.18906400 -0.34616900 -3.41583300  C 1.97455700 -1.38596600 -4.46962100  H 3.11192600 -0.54004500 -2.99241200  C 2.25962000 1.02183200 -4.01820900  H -2.08268300 -0.92447800 6.83727300  H -3.90917500 -0.94039400 8.50209400  H -6.21992100 -0.33187700 7.81661300  H -6.67542900 0.30655600 5.46088600  H -4.86173900 0.32918400 3.80098600  H -1.23180900 -0.47748000 4.69019800  H -3.32555900 -0.18764400 2.45635000  H -2.17003400 -0.31002900 -1.87789700  H -0.50123600 -0.31248200 -3.70543400  H 2.67016600 -0.48413600 -0.75828400  H 2.86050900 -1.39270600 -5.10350200  H 1.10161400 -1.11161400 -5.06029200  H 1.83610400 -2.35271700 -3.98981900  H 3.04354300 1.01210100 -4.77495100  H 2.50060200 1.74458100 -3.24204400  H 1.29527600 1.24665000 -4.47385100 | Charge = 0, Multiplicity = 1,  Imaginary freq = none  H -5.69720100 -1.43119800 0.01103700  H 6.28948600 -0.45942600 4.32209700  H 6.68382300 3.00145100 0.73697900  H -5.16816400 -0.47282900 5.57132000  H 1.59767400 -6.04622500 2.92326600  H -6.46535900 -1.05315700 4.47427300  H -1.61557500 6.49162000 1.08391700  H -4.62055200 3.07779700 -0.91042900  H 4.80491300 -4.80339100 2.34212300  H 7.02466000 -1.53578200 0.00014500  H 5.56345000 4.68482400 2.23706900  H -0.12605100 6.13590700 -0.81363700  H -6.47162700 2.97321300 2.23419700  H -1.65810200 -3.61921000 5.78217000  H 0.38246200 5.52813900 3.95200900  H 2.97795300 -3.68004000 5.28841900  H -5.89760100 -3.10506400 3.22456500  H 1.73232900 6.44683700 0.71539800  H -4.02198500 4.02684600 4.66756100  H 7.32936000 -0.98356700 2.95656900  H 6.54864400 -3.13730700 1.88020500  H 4.03465300 5.61603600 0.58666600  H 3.35594200 -5.14692500 4.32662500  H -2.30716900 -4.69240500 0.19504700  H -2.24464000 -5.09218900 4.94323300  H 4.81463600 3.99096000 3.71346300  H -4.10133500 -4.77776400 3.34913800  H -3.91426700 5.70145000 1.44976600  H 5.19532300 4.07906900 -0.92672700  H 2.35747900 -4.64132200 -0.32482700  H 7.47640700 0.70605300 1.11782200  H 2.99590100 -5.70956200 0.96809400  H -0.83794500 -6.03709100 3.23706900  H -6.74963800 -1.54704600 1.46077200  H -2.65231800 -5.73263200 1.61668600  H 4.15173900 2.95085700 -1.85451200  H -5.40766900 4.16743800 0.27709800  H -5.08988800 4.68151700 3.38189800  H 5.70786800 -1.46226500 -1.21673100  H -7.06071900 0.62046800 2.64075400  H 0.25592300 6.72290300 2.61974200  H -0.24302200 4.56306600 -1.66904600  O 4.38946900 0.55124900 -1.70788800  C 4.96140500 0.89662600 -0.68151800  N 4.96995800 2.16751900 -0.14018900  C 4.42706900 3.30005300 -0.85906000  N 3.24262500 3.86817000 -0.25624700  C 3.29268400 4.84333400 0.81455500  N 3.52640800 4.30325400 2.13917100  C 2.44438500 4.47871900 2.98311300  O 2.41377300 4.22510600 4.18069500  N 1.41635700 5.04003200 2.24263600  C 0.26826300 5.65584900 2.87548200  N -1.00356600 5.07169800 2.50438800  C -1.86316300 4.54728200 3.45650800  O -1.58468800 4.31005600 4.62439900  N -3.10199200 4.38970900 2.86613100  C -4.27557900 3.98445300 3.60805100  N -4.71282500 2.63365500 3.32471300  C -4.65427800 1.64042400 4.28743300  O -4.15602200 1.74341300 5.39903100  N -5.31084800 0.53209000 3.78810600  C -5.43138900 -0.69896700 4.53768900  N -4.54747400 -1.75631500 4.08724300  C -3.44139000 -2.15730500 4.81557000  O -3.03345800 -1.64957000 5.85042200  N -2.90620700 -3.27027100 4.18983900  C -1.87409500 -4.06800000 4.81244200  N -0.63110500 -4.12123500 4.06942600  C 0.55646600 -3.64839000 4.60121100  O 0.67029400 -2.93405700 5.58957500  N 1.59454700 -4.16168300 3.84389400  C 2.97041700 -4.12093700 4.29159100  N 3.84224900 -3.31552100 3.46508000  C 4.50441500 -2.21322700 3.97514600  O 4.33777900 -1.72405600 5.08520800  N 5.41971200 -1.79796300 3.02824700  C 5.53494000 -2.72470100 1.91857200  N 5.15900400 -2.21669800 0.61384500  C 5.96809000 -1.30740400 -0.16951400  N 5.73830000 0.08956200 0.12689300  C 6.39019000 0.82236400 1.19280300  N 5.95212000 0.51638300 2.54318500  C 6.31860600 -0.68885700 3.25661900  C 5.41293500 1.61336500 3.18471400  O 5.14144000 1.69499100 4.37695400  N 5.28669000 2.62652000 2.25553400  C 5.88809300 2.28891100 0.97884200  C 4.84582700 3.95340000 2.62434100  C 4.06361600 -2.86995800 0.08240800  O 3.63293500 -2.74383300 -1.05690500  N 3.55662900 -3.70796300 1.05873300  C 4.42521200 -3.78293500 2.22152300  C 2.57442700 -4.72276800 0.74035400  N 1.32118700 -4.57714500 1.44966900  C 1.13444000 -5.06061200 2.80559900  C -0.41464000 -5.05916500 2.98285000  N -0.90065500 -4.62262200 1.69000800  C 0.12038700 -4.39713700 0.78531200  O -0.01648500 -4.12990100 -0.40232800  C -2.28150400 -4.75619900 1.28295800  N -3.16148300 -3.72539300 1.79477300  C -3.75264100 -3.76449200 3.12130400  C -4.89552200 -2.70149600 3.04433200  N -4.79701200 -2.20942100 1.68554500  C -3.82862100 -2.85918100 0.94988300  O -3.62128900 -2.72423000 -0.25125200  C -5.74886500 -1.29802800 1.09190600  N -5.47958600 0.09523400 1.37056400  C -5.98552600 0.80040700 2.53482700  C -5.61396300 2.29183200 2.23839900  N -5.02476700 2.23150200 0.91608000  C -5.01429800 0.95412900 0.39446800  O -4.69159800 0.64166800 -0.74519900  C -4.65217900 3.38761100 0.13436100  N -3.35324300 3.93730600 0.46688800  C -3.15251700 4.91747300 1.51939300  C -1.69414800 5.41562100 1.27421700  N -1.27257200 4.66789000 0.10537800  C -2.27666800 3.86060400 -0.39459200  O -2.22957600 3.22279100 -1.44084200  C -0.13001600 5.04497700 -0.69805300  N 1.14456300 4.62961900 -0.15397600  C 1.82745100 5.36976700 0.89065000  C 1.99955900 3.79647600 -0.85525900  O 1.71426600 3.14725800 -1.85391500  C 4.21741100 1.27288800 7.79968900  C 5.03387800 1.44274000 8.91504700  C 4.48685400 1.45764700 10.20392100  C 3.10728700 1.30649400 10.36511000  C 2.28657500 1.13713800 9.25013300  C 2.82205800 1.10585400 7.94659500  C 2.32008200 0.63438800 5.55191700  C 0.27296800 -0.67464000 -2.79831300  C -1.93560100 -0.16156500 -1.68962500  N -0.49245500 -0.37578800 -1.59337400  O -0.65098400 0.76931300 5.33258900  O -0.59196700 0.42763000 3.13995500  C 0.04587700 0.56260200 4.36287400  C 1.52104600 0.44982400 4.35527400  N 2.18426000 0.18722900 3.24959900  C 1.56304300 -0.39184600 -0.31112500  C 2.21445700 -0.25350200 0.88933500  C 1.51415400 0.03129700 2.07819400  C 0.11176400 0.16620300 1.99077000  C -0.57803600 0.04710100 0.79788900  C 0.14214400 -0.24063900 -0.39577400  H -2.21041500 0.85721900 -1.40313100  H 3.37548300 0.51968100 5.33319700  H 4.65977300 1.28830400 6.80814600  H 0.82472800 -1.61697400 -2.69790800  H 2.66914400 1.32195300 11.35940600  H 2.15704500 -0.61085400 -1.18521400  H -1.65236700 0.17123200 0.80472500  H -2.24639400 -0.31650700 -2.72354600  H 1.21371200 1.01963500 9.38236000  H 0.99170100 0.12124300 -3.03177900  H -0.41372500 -0.77213300 -3.63854900  H 5.12859900 1.59335200 11.06986900  H 6.10449400 1.57274800 8.77990700  H 3.28906000 -0.36450200 0.95551700  H -2.48968100 -0.86595000 -1.06342100  C 1.91384000 0.91015600 6.81255000  H 0.85358900 0.98185400 7.03036200 | Charge = 1, Multiplicity = 1,  Imaginary freq = none  H 6.13147500 2.45898000 -1.69614300  H -6.48469900 -0.04594800 0.08952800  H -4.91356800 -3.96298300 -2.39629600  H 5.05737600 2.12637500 3.86209500  H -2.11468800 5.96079900 -0.53748400  H 6.33288100 2.87061400 2.84528600  H 3.41871900 -5.79721400 0.27765600  H 6.24360800 -2.27181300 -1.97196100  H -5.01902700 4.21288800 -1.84818000  H -5.60870000 0.22938100 -4.28226800  H -3.87175600 -4.90542700 -0.25135400  H 2.34750200 -6.11013400 -1.90850700  H 7.57775100 -1.28308000 1.31971200  H 0.76628800 3.81550700 2.90205400  H 0.83166400 -4.30807200 2.37463100  H -3.65960800 3.48048000 1.51416200  H 5.50168700 4.54178600 1.20310200  H 0.27871900 -6.24380600 -0.60616000  H 5.08569100 -2.56657200 3.56009600  H -6.97191400 0.06204900 -1.63011200  H -6.16222400 2.20717500 -2.74788600  H -2.02703000 -5.84610400 -1.36452200  H -3.97009500 4.86654800 0.42089200  H 2.38816400 5.24737100 -2.42108100  H 1.20892300 5.44303500 2.29525000  H -3.56128700 -3.68508500 1.02662000  H 3.30709100 5.66670100 1.10274900  H 5.59291100 -4.66637400 0.58895400  H -2.96185400 -4.99311100 -3.50733500  H -2.01405600 4.48188800 -3.86421400  H -6.07262200 -1.87844700 -3.05624000  H -3.06378100 5.42101300 -2.75367900  H 0.18132000 6.23805700 0.28673800  H 6.91937200 3.02433100 -0.18689200  H 2.22017500 6.35775300 -1.02198700  H -1.90942200 -3.88391800 -4.44521000  H 6.99078700 -3.01912900 -0.52243200  H 6.39734400 -3.13788600 2.47701400  H -3.93884700 0.31480800 -4.93360900  H 7.55770500 1.17601500 1.35897000  H 1.27372600 -5.80617600 1.49382900  H 2.49388900 -4.77734400 -3.10178900  O -2.27553500 -1.42686500 -4.59304000  C -3.12702800 -1.75371000 -3.77668600  N -3.21911100 -2.97598200 -3.14174200  C -2.35085500 -4.08490900 -3.46903000  N -1.25898700 -4.28750400 -2.54425000  C -1.42286200 -4.93901100 -1.26030300  N -1.96825000 -4.09339500 -0.21736300  C -1.04491700 -3.81452700 0.77580800  O -1.25993600 -3.16855400 1.79675300  N 0.15073300 -4.42180200 0.43116700  C 1.16760300 -4.71549600 1.42113200  N 2.46087100 -4.12622800 1.13919000  C 3.13488100 -3.43398700 2.13586600  O 2.64566400 -3.00466300 3.17144000  N 4.46486400 -3.36138600 1.77237300  C 5.43989500 -2.61113700 2.53008700  N 5.62068900 -1.24589800 2.07919700  C 5.17332500 -0.18089100 2.84123100  O 4.48127900 -0.25343700 3.84628600  N 5.69413100 0.97488400 2.29285200  C 5.41277800 2.27973600 2.84298200  N 4.39727700 3.02832800 2.13333800  C 3.06715100 3.01858000 2.50133000  O 2.58743300 2.42102800 3.45399200  N 2.37562500 3.84665100 1.62357700  C 1.09112700 4.38344700 2.03053300  N 0.05734100 4.27467700 1.02480400  C -1.13804600 3.62435400 1.27848400  O -1.34837000 2.82470300 2.18462800  N -2.06359600 4.05927700 0.34422800  C -3.48825700 3.88735300 0.51778000  N -4.09921300 2.96698400 -0.41970500  C -4.73210800 1.82931400 0.01074500  O -4.73255600 1.39998900 1.16717600  N -5.37073200 1.25073200 -1.05977800  C -5.19175500 2.01120000 -2.28343000  N -4.26991700 1.43555600 -3.24674200  C -4.54386000 0.25245400 -4.02878400  N -4.19250100 -0.97848500 -3.35267900  C -5.12589200 -1.73044300 -2.52852000  N -5.37124600 -1.18555500 -1.20888000  C -6.12842800 0.01882400 -0.93831600  C -4.70683300 -1.88219800 -0.22473000  O -4.74857700 -1.64185600 0.98189500  N -4.00189000 -2.90275400 -0.81731800  C -4.33882200 -3.04648200 -2.23386700  C -3.39305700 -3.94744200 -0.01778300  C -3.21613200 2.27551500 -3.54269100  O -2.36984400 2.09414900 -4.41059200  N -3.30107700 3.36824600 -2.70589100  C -4.42962500 3.29251700 -1.80520400  C -2.44612800 4.52325800 -2.86430100  N -1.34716100 4.58670400 -1.92689000  C -1.51389300 5.04589200 -0.56254000  C -0.05087300 5.22745600 -0.06551100  N 0.75050800 4.90108500 -1.22838000  C -0.02437300 4.57150800 -2.32648800  O 0.38939400 4.33902600 -3.45603300  C 2.13046800 5.32014900 -1.36461000  N 3.07606200 4.51291900 -0.63115900  C 3.29925500 4.61677800 0.79158400  C 4.65369200 3.87987000 0.99905400  N 4.84400700 3.19833700 -0.27703100  C 3.99345600 3.68773100 -1.25286400  O 4.05642200 3.46213400 -2.45554300  C 6.06190500 2.48782200 -0.60833700  N 6.08082200 1.12222500 -0.13209800  C 6.57788200 0.72310900 1.17598800  C 6.60407400 -0.83981200 1.08760700  N 6.25431400 -1.09631600 -0.29410300  C 5.98275500 0.05461500 -1.00307500  O 5.73570200 0.12108000 -2.20119400  C 6.16374000 -2.40507800 -0.89298700  N 4.91190200 -3.09433700 -0.63144500  C 4.73354600 -3.99388700 0.50388000  C 3.38885700 -4.70767400 0.17269800  N 3.14310300 -4.33714100 -1.20129600  C 4.03153200 -3.38586100 -1.66013300  O 4.05359800 -2.91151700 -2.79005700  C 2.22900000 -5.03317500 -2.07607200  N 0.83813400 -4.68274200 -1.89371200  C 0.04164700 -5.19204100 -0.79733800  C 0.06501900 -4.18693900 -2.92766600  O 0.47854400 -3.77285400 -4.00330200  C -4.84084100 -0.55175700 6.28475300  C -5.80203700 -0.63755400 7.28559900  C -7.16533500 -0.60882100 6.96373400  C -7.56218400 -0.49216500 5.62890300  C -6.60233700 -0.40462400 4.62367900  C -5.22453300 -0.43502500 4.93024500  C -2.92492100 -0.39362700 3.92244400  C 0.88954400 0.70919100 -3.90049000  C 2.78536100 0.46197300 -2.29652500  N 1.34375500 0.57574900 -2.51508300  O -0.11419000 -0.54526800 4.24270100  O 0.31030500 -0.22786000 2.08875900  C -0.55995800 -0.37346200 3.13054700  C -2.01000100 -0.29281300 2.82689100  N -2.36512400 -0.12583700 1.55938600  C -0.95924200 0.33567500 -1.81466200  C -1.89364200 0.16767500 -0.82244000  C -1.48895200 -0.00212200 0.51521400  C -0.10918300 -0.03145400 0.79796000  C 0.84783800 0.12693600 -0.17783500  C 0.44591400 0.35151200 -1.52468500  H 3.12980800 -0.57089800 -2.42066200  H -2.43840400 -0.52060500 4.88240200  H -3.78984800 -0.57129700 6.55450200  H 0.20240400 1.55426300 -4.00940600  H -8.61737600 -0.46900000 5.37277200  H -1.30790400 0.45009100 -2.83088000  H 1.88678800 0.07841800 0.11695900  H 3.30847200 1.10096400 -3.00443000  H -6.90901500 -0.31407300 3.58490500  H 0.38784700 -0.20303700 -4.24626400  H 1.75905300 0.88692800 -4.53258900  H -7.91071000 -0.67630400 7.75080400  H -5.49182500 -0.72552400 8.32275400  H -2.94365000 0.15681500 -1.08354500  H 3.05974800 0.81395300 -1.30306800  C -4.28162600 -0.34169700 3.82703500  H -4.74239100 -0.22533600 2.85188800  H -3.36684900 -0.05083500 1.34159100 |
